# Roux-en-Y gastric bypass-associated fecal tyramine promotes colon cancer risk via increased DNA damage, cell proliferation, and inflammation

**DOI:** 10.1186/s40168-025-02049-2

**Published:** 2025-02-28

**Authors:** Maria Glymenaki, Sophie Curio, Smeeta Shrestha, Qi Zhong, Laura Rushton, Rachael Barry, Mona El-Bahrawy, Julian R. Marchesi, Yulan Wang, Nigel J. Gooderham, Nadia Guerra, Jia V. Li

**Affiliations:** 1 Division of Digestive Diseases, Department of Metabolism, Digestion and Reproduction, Imperial College London, London, UK; 2 Department of Life Sciences, Imperial College London, London, UK; 3 Singapore Phenome Center, Lee Kong Chian School of Medicine, Nanyang Technological University, Singapore, 308232 Singapore; 4 The University of Queensland Frazer Institute, The University of Queensland, Woolloongabba, QLD 4102 Australia; 5 Department for Environment Food and Rural Affairs, London, UK

**Keywords:** Colon cancer risk, Bariatric surgery, Prevention, Inflammatory bowel disease (IBD), Host-microbial interaction

## Abstract

**Background:**

Fecal abundances of *Enterobacteriaceae* and *Enterococcaceae* are elevated in patients following Roux-en-Y gastric bypass (RYGB) surgery. Concurrently, fecal concentrations of tyramine, derived from gut bacterial metabolism of tyrosine and/or food, increased post-RYGB. Furthermore, emerging evidence suggests that RYGB is associated with increased colorectal cancer (CRC) risk. However, the causal link between RYGB-associated microbial metabolites and CRC risk remains unclear. Hence, this study investigated the tyrosine metabolism of *Enterobacteriaceae* and *Enterococcaceae* strains isolated from patients post-RYGB and explored the causal effects of tyramine on the CRC risk and tumorigenesis using both human colonic cancer cell line (HCT 116) and wild-type and *Apc*^*Min/*+^ mice.

**Results:**

We isolated 31 bacterial isolates belonging to *Enterobacteriaceae* and *Enterococcaceae* families from the feces of patients with RYGB surgery. By culturing the isolates in tyrosine-supplemented medium, we found that *Citrobacter* produced phenol as a main product of tyrosine, whereas *Enterobacter* and *Klebsiella* produced 4-hydroxyphenylacetate, *Escherichia* produced 4-hydroxyphenyllactate and 4-hydroxyphenylpyruvate, and *Enterococcus* and two *Klebsiella* isolates produced tyramine. These observations suggested the gut bacterial contribution to increased fecal concentrations of tyramine post-RYGB. We subsequently evaluated the impact of tyramine on CRC risk and development. Tyramine induced necrosis and promoted cell proliferation and DNA damage of HCT 116 cells. Daily oral administration of tyramine for 49 days to wild-type mice resulted in visible adenomas in 5 out of 12 mice, accompanied by significantly enhanced DNA damage (γH2AX +) and an increased trend of cell proliferation (Ki67 +) in the ileum, along with an upregulated expression of the cell division cycle gene (*Cdc34b*) in the colon. To evaluate the impact of tyramine on intestinal tumor growth, we treated *Apc*^*Min/*+^ mice with the same doses of tyramine and duration. These mice showed larger colonic tumor size and increased intestinal cell proliferation and inflammation (e.g., increased mRNA expression of *IL-17A* and higher number of Ly6G + neutrophils) compared to water-treated *Apc*^*Min/*+^ control mice.

**Conclusions:**

Our results collectively suggested that RYGB-associated fecal bacteria could contribute to tyramine production and tyramine increased CRC risk by increasing DNA damage, cell proliferation, and pro-inflammatory responses of the gut. Monitoring and modulating tyramine concentrations in high-risk individuals could aid CRC prognosis and management.

Video Abstract

**Supplementary Information:**

The online version contains supplementary material available at 10.1186/s40168-025-02049-2.

## Introduction

Bariatric surgery, including Sleeve Gastrectomy (SG) and Roux-en-Y gastric bypass (RYGB), is an effective surgical treatment for weight loss and improving body weight-associated conditions, such as hypertension, diabetes, arthritis, and apnea [1]. Bariatric surgery has been shown to reduce various cancer risks with the most positive impact on post-menopausal breast and endometrial cancer [2]. While the association between the surgery and colon cancer is inconclusive, emerging evidence from large population cohort studies has suggested an increased colon cancer risk [3–5]. This increased risk is further supported by observations that colorectal epithelial cell proliferation and expression of tumorigenic molecules, e.g. Cyclooxygenase-1 (COX-1) and COX-2, were increased [6].

The gut microbiome, including both microbial composition and metabolism, has been found to be significantly altered post-RYGB surgery [7, 8]. Increased relative abundance of *Enterobacteriaceae* and *Enterococcaceae* families were found post-operation in both rodent models [9–11] and patients [8, 12–14]. Additionally, increased fecal excretion of tyramine, phenylacetate, and putrescine post-RYGB surgery suggested a shift from protein degradation to protein fermentation [8, 9], which is associated with colorectal cancer (CRC) [15–17]. Furthermore, fecal abundances of *Enterobacteriaceae* and *Enterococcaceae* were elevated in patients with inflammatory bowel disease (IBD), which increases CRC risk [13, 18]. Fecal tyramine levels were found to be higher in patients with ulcerative colitis (UC) [19] and associated with Crohn’s Disease (CD) severity assessed by Montreal S score [20]. Moreover, tyramine was associated with intestinal adenoma and carcinoma using a microbiome metabolic modeling method [21]. These observations collectively led us to conclude that there may be a potential causal link between tyramine and increased CRC risk.

Tyramine is a metabolic by-product of aromatic amino acid tyrosine by the bacterial tyrosine decarboxylase [22, 23], which is widely reported in *Lactobacillus* species [24] and *Enterococcus faecalis* (*Enterococcaceae* family) [25]. The latter has been shown to be associated with IBD and CRC [26]. However, tyramine production and tyrosine degradation have not been fully investigated in RYGB-associated gut commensal bacterial species. In addition to the gut microbiota as a source, tyramine can be found in high concentrations in foods, such as processed meat, which is known to increase CRC risk [22]. A previous study reported that tyramine altered the expression profile of genes involved in DNA damage and repair in a colonic epithelial cell line [27]. However, the role of tyramine in modulating CRC risk and tumorigenesis has not been investigated. Given that IBD and bariatric surgery are associated with increased CRC risk, there is a critical need to understand the consequences of elevated fecal tyramine to mitigate the long-term health risks.

In this study, we explored the metabolic activities of RYGB-associated gut microbiota with a focus on the tyrosine degradation activity in *Enterobacteriaceae* and *Enterococcaceae* isolates. We next investigated the function of tyramine in modulating CRC risk factors including DNA damage, cell proliferation, and inflammation using both HCT 116, a human colonic epithelial cell line, and wild-type C57BL/6J mice. The effect of tyramine on tumor development was explored using a genetically altered *Apc*^*Min/*+^ mouse model.

## Material and methods

### Bacterial isolation

Feces were collected from two patients at 2 years post-RYGB surgery from Hammersmith Hospital, London, UK (Ethics reference: 13/LO/1510). Patient fecal samples were mixed with 8% v/v sterile dimethyl sulfoxide (DMSO) as a cryoprotectant and stored at − 80 °C prior to bacterial isolation. A non-selective nutrient-rich yeast casitone fatty acid (YCFA) medium [28] and a MacConkey medium (Merck, Germany) prepared according to the manufacturer’s guidelines to select for *Enterobacteriaceae* were used to isolate 31 bacteria under aerobic conditions at 37 °C. Crystal violet (0.001 g/L) was added to the MacConkey medium as an additional inhibitor of gram-positive bacteria.

### Bacterial DNA extraction, 16S rRNA gene-based polymerase chain reaction (PCR), and random amplified polymorphic DNA analysis (RAPD)

Bacterial cell pellets were collected by centrifuging the culture for 10 min at 1400 × *g* and resuspended in 600 µl of guanidine isothiocyanate, a protein denaturant. The suspension was then mixed with 1 g of 0.1 mm high-impact zirconium beads (Benchmark scientific) and homogenized for 30 s using Maxwell® Research Instrument for DNA extraction (Promega) with a Maxwell® 16 Tissue DNA Purification Kit (Promega). The extracted gDNA was quantified using a Quibit™ 3.0 Fluorometer with Qubit™ dsDNA BR (Broad range) assay kit (Thermo fisher Scientific).

To obtain a preliminary identification of bacterial genera, 16S rRNA gene-based Polymerase Chain Reaction (PCR) was carried out. 27F (5′-AGA GTT TGA TC(AC) TGG CTC AG-3′) and 1492R (5′-GGT TAC CTT GTT ACG ACT T-3′) are used to amplify complete 16S rRNA gene (~ 1500 bp). The volume of the PCR reaction was 25 µl, containing 12.5 µl of DreamTaq (2X), 1 µl of primers (1:1, 10 pmol/µl), 9.5 µl of molecular grade dH_2_O, and 2 µl of DNA template. The PCR thermocycler setting consisted of 1 cycle of initial denaturation at 95 °C for 2 min, 35 cycles of denaturation at 94 °C for 30 s, annealing at 52 °C for 30 s and extension at 72 °C for 90 s, and a cycle of final extension at 72 °C for 5 min. PCR products were cleaned using Monarch PCR & DNA cleanup kit (New England Biolabs) following the manufacturer’s instructions. The purified PCR products were sanger sequenced (Eurofins Genomics, Germany), and the data was compared with the Reference Sequence (RefSeq) database at the National Center for Biotechnology Information (NCBI) using the basic local alignment search tool (BLAST). The top hit by nucleotide BLAST was used as a preliminary identification of genera.

Random amplified polymorphic DNA analysis (RAPD) was utilized as previously described [29] to genotype and differentiate the collection of bacterial isolates. The PCR reaction volume was 25 µl consisting of 12.5 µl of DreamTaq (2X), 4 µl of 10 µM primer 272 (5′-AGCGGGCCAA-3′) [29], 6.5 µl of molecular grade dH_2_O, and 2 µl of DNA template (~ 20 ng). The PCR thermocycler setting consisted of 1 cycle at 94 °C for 5 min, 4 cycles of 5 min at each temperature (36 °C, 72 °C, and 94 °C), 30 cycles of 1 min at 94 °C, 1 min at 36 °C and 2 min at 72 °C, followed by 10 min at 72 °C. The PCR products were visualized using a 4200 TapeStation System (Agilent Technologies Ltd., UK) with D1000 ScreenTape and reagents, according to the manufacturer’s protocol. A visual assessment of banding profiles was carried out to differentiate isolates, identify RAPD profile types, and identify duplicates within a group.

### Bacterial culture with a tyrosine-supplemented medium

Bacterial isolates were revived from storage at − 80 °C by culturing on fastidious anaerobe agar (FAA) plates aerobically at 37 °C for 24 h. Three single colonies from each bacterial isolate were inoculated into 3 mL tryptic soy broth (TSB) for 24-h incubation at 37 °C. Each isolate was then washed with phosphate-buffered saline (PBS) and seeded in a 96-well plate containing a defined medium base [30] supplemented with tyrosine or tyrosine and glucose. Glucose was used to investigate whether the utilization of tyrosine by bacterial isolates differs in the presence or absence of glucose as an additional carbon source. In the colonic environment, it is expected that multiple carbon sources are available for bacterial growth. In brief, for 500 mL of the defined medium base, 3.5 g K_2_HPO_4_, 1.5 g KH_2_PO_4_, 0.5 g (NH_4_)_2_SO_4_, 0.05 g MgSO_4_∙7H_2_O, 0.05 g ferric citrate, 15 mg L-tryptophan, 15 mg L-histidine, 15 mg L-isoleucine, 15 mg nicotinamide and 25 mL 1 M sodium fumarate were added to water. The tyrosine-supplemented medium consisted of 4 mL of 2.5 M L-tyrosine and 46 mL of the defined medium base and the tyrosine and glucose-supplemented medium contained 0.5 g/L glucose. A total of 200 µl of the medium was transferred into each well of a 96-well plate before adding 50 µl of PBS containing the washed bacterial isolates. For the aerobic condition, plates were sealed with gas-permeable sealing films (Sigma-Aldrich) and incubated at 37 °C in an aerobic shaker at 80 rpm for 24 h to ensure good aeration. For the anaerobic condition, plates were sealed with polymerase chain reaction (PCR) plate sealing films (Fisher Scientific) and incubated in an anaerobic chamber at 37 °C, 70% humidity for 24 h for statical incubation. Negative controls were the wells containing the medium in the absence of bacteria and subjected to each culture condition. Each bacterial isolate was cultured in 3 wells per culture condition.

### ^1^H nuclear magnetic resonance (NMR) spectroscopic analysis of the bacterial culture medium

^1^H NMR spectroscopy was used to detect the bacterial metabolites of tyrosine and glucose. Triplicates of each isolate were combined and centrifuged at 20,000 × *g* at 4 °C for 10 min, and 540 μl of supernatant was mixed with 60 μl of 1.5 M phosphate buffer (pH = 7.4) containing 2 mM sodium azide (NaN_3_) and 5.8 mM 3-(trimethylsilyl)-[2,2,3,3-^2^H_4_]propionic acid sodium salt (TSP) [31]. The resulting mixture was vortexed and centrifuged at 20,000 × *g* at 4 °C for another 10 min. A total of 575 μl supernatant was transferred into an NMR tube with an outer diameter of 5 mm for ^1^H NMR spectra acquisition using a Bruker 600 MHz spectrometer (Bruker Corporation, Rheinstetten, Germany) at a proton frequency of 600.13 MHZ at 300 K. Standard NMR pulse sequence (recycle delay [RD]−90°-t_1_−90°-t_m_−90°-acquisition) with mixing time (*t*_m_) of 100 ms were set to acquire 1-dimensional ^1^H NMR spectral data. 90° pulse length was set to about 10 μs, and RD of 4 s was set to achieve water suppression [31]. A total of 16 scans were collected into 64 K data points with a spectral width of 20 ppm.

The spectra were automatically phased, baseline-corrected, and calibrated to the TSP singlet at δ ^1^H 0 in Topspin software (Bruker) and imported into MATLAB (R2018a). Metabolite identification was carried out using statistical total correlation spectroscopy [32] (STOCSY), human metabolome database [33], Chenomx NMR suite (Version 10.1), and an internal database of standard compounds. The relative concentrations of each metabolite were represented by the areas under the selected peak of a given metabolite (Table S1) and calculated using MATLAB (R2018a). Ratios of relative concentrations of metabolites in bacterial media to the negative control medium without bacteria were calculated as fold changes.

### HCT 116 cell culture and treatment

The HCT 116 cell line, a human colorectal adenocarcinoma adherent cell line, was obtained from the American Type Culture Collection (ATCC, RRID: CVCL_0291, LGC Prochem). Cells were routinely cultured in RPMI 1640 medium (Gibco) supplemented with 10% fetal bovine serum (FBS), 100 units/mL penicillin, 100 μg/mL streptomycin, and 2 mmol/L L-glutamine (Gibco). The cells were passaged every 2–3 days and passages 3–7 were used in the current study.

Cells were maintained in a culture medium supplemented with 5% dextran-coated charcoal-stripped FBS for 72 h to remove hormones and growth factors before any treatment. The cells were seeded and treated with various concentrations of tyramine dissolved in buffered water (0.2 mM to 6.4 mM, pH = 7.01) for 24, 48, or 72 h. Specifically, 20,000 cells per well were used for cell viability, 40,000 for genotoxicity, and 100,000 cells for flow cytometry assays.

### Cytotoxicity assay

To assess cell viability, the alamarBlue ready-to-use reagent (Invitrogen) was added at a volume of 1:10 in cell culture media. The alamarBlue cell viability reagent utilizes the mitochondrial metabolic reducing power of cells to convert resazurin, the active ingredient of alamarBlue, to resorufin thus assessing cell viability. Fluorescence was read using a Fluostar plate reader (BMG Labtech, Ortenberg, Germany) at excitation 560 nm/emission 590 nm. Etoposide at a final concentration of 125 nM was used as a positive control.

### Micronucleus (MN) assay

After 48 h of treatment, cells were harvested and resuspended in 500 µL 2% v/v pluronic acid. Eighty microliters of cell suspension were loaded in a cytospin for 5 min at 600 rpm to attach the cells on a glass slide. Slides were air dried and fixed in 100% methanol for 10 min. They were washed in PBS, and stained with acridine orange (0.1 mg/mL) for 60 s followed by two washes in PBS as previously described [34]. They were left to air dry in the dark, and MN scoring was performed blind in 2000 cells per sample and three biological replicates per treatment.

### Cell staining for apoptosis and necrosis using flow cytometry

Cells were harvested after treatment with 1 ml Cell Dissociation Buffer (Gibco), washed twice with Cell Staining Buffer (Biolegend), and resuspended in 100 µl Annexin V Binding Buffer (Biolegend). PE Annexin V (Biolegend) was added, and cells were incubated for 15 min at room temperature in the dark. After Helix NP Blue (5 nM) (Biolegend) was added, cells were acquired in a BD LSR Fortessa flow cytometer and analyzed with FlowJo version 10.7.1 or above (RRID:SCR_008520, BD).

### Cell staining for cell cycle analysis using flow cytometry

Cells were detached using the Cell Dissociation Buffer (Gibco), washed with PBS, and fixed with chilled 70% v/v ethanol at − 20 °C for 1 h. The fixed cells were subsequently washed with PBS and Cell Staining Buffer (Biolegend). FxCycle Violet stain (1000 ng/ml) (Invitrogen) was added followed by a 30-min incubation at 4 °C before analysis using a BD LSR Fortessa flow cytometer. Analysis was performed in FlowJo version 10.7.1 or above (BD).

### Cell staining for proliferation and DNA damage analysis using flow cytometry

Cells were treated with the Cell Dissociation Buffer (Gibco), washed with PBS, and incubated with the Human BD Fc Block (BD) and the LIVE/DEAD Fixable Far Red Dead Cell Stain Kit (Invitrogen) for 20 min at 4 °C. They were subsequently washed with 0.5% PBS-BSA, and fixed and permeabilized using the Foxp3/Transcription Factor Staining Buffer Set (eBioscience) to stain for intracellular epitopes including Ki-67 eFluor 450 (1.33 μg/ml, clone SolA15, eBioscience) and Alexa Fluor 488 Mouse anti-H2AX (pS139) (dilution 1:400, clone N1-431, RRID: AB_1645352, BD). The relevant fluorescence-minus-one, single-stained, and unstained cell suspensions were used as controls. Samples were acquired in a BD LSRFortessa flow cytometer and analyzed with FlowJo version 10.7.1 or above (BD).

### Animal model and experiment design

C57BL/6J-*Apc*^*Min*^/J mice (*Apc*^*Min/*+^*)*, a model of multiple intestinal adenomas [35, 36] (RRID:IMSR_JAX:002020) and wild-type (WT) littermates C57BL/6J (RRID:IMSR_JAX:000664) were purchased from the Jackson Laboratory (Bar Harbor, ME USA). The *Apc*^*Min/*+^ strain was maintained by breeding heterozygous *Apc*^*Min/*+^ males to WT females. Animals were genotyped using the primers *Apc*-wild-type 5′-GCC ATC CCT TCA CGT TAG-3′, *Apc*-common antisense 5′- TTC CAC TTT GGC ATA AGG C-3′ and *Apc*-mutant 5′-TTC TGA GAA AGA CAG AAG TTA-3′ as previously described [37]. Animals were kept under specific, pathogen-free conditions at the Central Biomedical Services (CBS) facility at Imperial College London in a 12-h light/dark circle. The health status of the animals was routinely monitored throughout the study. All experimental procedures in mice were performed in accordance with the ethical regulation for animal use in research issued by the UK Home Office under the Animals (Scientific Procedures) Act 1986. The study received ethical approval (Project License: 9718F9C8) from the UK Home Office, and the CBS facility at Imperial College also approved all the study protocols performed.

*Apc*^*Min/*+^ and WT mice at 6 weeks of age were orally gavaged daily with 200 µl of sterile-filtered 3.2 mM tyramine (Sigma-Aldrich) solution at pH = 7.01 or water for 7 weeks. We culminated the experiment at 13 weeks of age of mice, which represents an early stage of disease based on the previous survival analysis in *Apc*^*Min/*+^ mice that showed a drop in survival after 18–20 weeks of age [38]. Food consumption, water intake, and body weight were monitored weekly throughout the study. Animals were blind scored for the development of macroscopic signs of disease appearance (Supplementary Table S2). Prior to the commencing of the study, and at 4 and 7 weeks into the study, mice were put in metabolic cages overnight with ad libitum access to food and water for the collection of urine and feces. Animals were singly housed to avoid cage-effects on microbial composition and metabolism throughout the study.

### Mouse tissue and biofluid sample collection

Tissue samples were collected at the end of the study. After the gut length measurement, the small intestine was divided into three parts representing duodenum, jejunum, and ileum. Following the removal of the caecum, the colon was isolated. Luminal contents were collected from each part and were snap-frozen in liquid nitrogen. Gut tissue segments were opened longitudinally and washed with sterile PBS. Tumors were counted and tumor size was recorded. Gut tissue samples were collected for RNA analysis, and immunohistochemistry, and were also snap-frozen for future studies. The liver and spleen were also collected and weighed. Blood was collected into Microvette EDTA 200 K3E tubes (Sarsted) for hematocrit measurement using a hematology XN-L analyzer (Sysmex). Biofluid samples were stored in a − 80 °C freezer pending analysis. Researchers were blinded during tissue harvesting regarding the treatment regime of the animals.

### Immunohistochemistry

Immunohistochemistry analysis of the tissue sections was carried out by the Histopathology Lab at the Barts Cancer Institute. Paraffin-embedded sections were stained for the presence of TNF-a (ThermoFisher Scientific), β-catenin (clone CAT-5H10) (ThermoFisher Scientific), Ki-67 (clone SolA15) (eBioscience), phospho-Histone H2A.X (clone 20E3) (Cell Signaling Technology), and Ly6G (clone RB6-8C5) (eBioscience). Staining with relevant isotype controls was performed to confirm there was no non-specific binding. Images of the sections were obtained using a NanoZoomer S210 slide scanner (Hamamatsu) and visualized using NDP.view2 software (Hamamatsu). Image analysis was performed using ImageJ (RRID:SCR_003070, http://rsb.info.nih.gov/ij). All slides were blinded prior to analysis and three sections per slide were used.

### RNA extraction

HCT 116 cells (~ 100,000 cells) were seeded and treated with 1.2 mM or 1.6 mM tyramine or left untreated for 24 h. Lysis buffer (Qiagen) was directly added to the cells and RNA was extracted using the RNeasy Mini Kit (Qiagen). Tissue from animals was stabilized in RNAprotect Tissue Reagent (Qiagen) and RNA was purified using the miRNeasy Micro Kit (Qiagen). RNA was treated with RNAse-Free DNase Set (Qiagen) followed by RNA clean-up in both procedures. The total RNA concentration, the RNA Integrity Number (RIN), and the 28S/18S ratio were measured using an Agilent 2100 Bioanalyzer (Agilent).

### RNA sequencing

RNA sequencing analysis was performed by Imperial BRC Genomics Facility (Imperial College London). Library was prepared using total RNA for cells and polyA mRNA for tissue samples. High-throughput sequencing was run in a HiSeq 4000 (Illumina) to obtain 75 bp sequencing paired-end reads. Sequencing data were mapped against a reference transcriptome with STAR alignment, followed by quantification using RSEM as detailed in alternate protocol 7 of the STAR (RRID:SCR_004463) software package [39]. Gene counts were generated using featureCounts [40] and subsequent analysis was performed using DESeq2 (RRID:SCR_000154) pipeline [41] following the RNA-Seq workflow [42]. The DESeqDataSet object in cell RNA-seq data was built using the design formula ~ *Tyramine_treatment* (i.e., untreated, 1.2 mM tyramine, or 1.6 mM tyramine) to determine the effect of tyramine treatment. For the mouse gut tissue RNA-seq data, genotype (*Apc*^*Min/*+^ or WT) and treatment (tyramine or water) categories were combined in a new group, as we focused on the effect of tyramine treatment in *Apc*^*Min/*+^ and WT groups, separately. The new group contained the following levels tyramine-treated *Apc*^*Min/*+^, water-treated *Apc*^*Min/*+^, tyramine-treated WT, and water-treated WT. The design formula used was as follows ~ *Gender* + *Parental* + *group.* Pre-filtering encompassed removing rows with less than 10 counts across all samples. We considered a gene to be differentially expressed if the false discovery rate (FDR) < 0.05, and the absolute log2 fold change (LFC) > 1. Gene ontology (GO) analysis was performed using goseq package, as it takes into account gene length bias [43]. Gene Set Enrichment Analysis (GSEA) was applied to identify gene sets correlated with the phenotypic class of interest based on a ranked gene list [44]. Gage package for gene set or pathway analysis was also used [45]. FDR correction has been applied to GO terms and gene sets identified by GSEA and Gage package. Ingenuity Pathway analysis (IPA, RRID:SCR_008653, Qiagen) was applied for pathway analysis. Graphs were plotted using the R ggplot (RRID:SCR_014601) package [46].

### Statistical analysis

Statistical analysis was performed using Prism software (GraphPad, RRID:SCR_002798) and R (version 4.2.1). Data were assessed for normal distribution using the Kolmogorov–Smirnov normality test and the relevant statistic test was applied. No samples or animals were excluded from the analyses. Normally distributed data were analyzed by one-way ANOVA with Dunnett’s or Tukey’s multiple comparisons test or unpaired *t*-test test as appropriate to the number of comparisons being made. Data that did not exhibit a normal distribution were analyzed using the nonparametric Kruskal–Wallis test with Dunn’s multiple comparisons test or Mann–Whitney test as appropriate to the number of comparisons being made. FDR correction was applied for multiple Mann–Whitney test comparisons. For grouped data, a two-way ANOVA or mixed effects model followed by Tukey’s multiple comparisons test was used. Correlation analysis was performed using the Spearman correlation coefficient. Contingency table analysis using Fisher’s exact was applied. *P* < 0.05 was considered as statistically significant.

## Results

### Tyrosine degradation varied in the fecal bacterial isolates from patients with RYGB

Our previous study showed that bacterial tyrosine metabolism was significantly altered in patients following RYGB surgery [8] and the fecal tyrosine degradation pathway was upregulated in patients with CRC [17]. These observations have led us to further investigate if these RYGB-associated bacterial species from *Enterobacteriaceae* and *Enterococcaceae* families metabolize tyrosine. A total of 31 bacterial isolates were obtained from the fecal samples of two patients post-RYGB and genotyped using 16S rRNA gene-based PCR and RAPD analysis. The RAPD types, namely, Citrobacter A and B, Enterobac A, B and C, Enteroc A, B, C, D and E, Esch A, B, C, D and E, and Kleb A, B and C, were identified (Table 1).

**Table 1:** Bacterial genus and random amplified polymorphic DNA (RAPD) types of 31 isolates from the fecal samples of patients 2 years post-RYGB surgery

No	Genus	RAPD type
1	*Citrobacter*	Citro A
2	*Citrobacter*	Citro B
3	*Enterobacter*	Enterobac A
4	*Enterobacter*	Enterobac A
5	*Enterobacter*	Enterobac B
6	*Enterobacter*	Enterobac B
7	*Enterobacter*	Enterobac C
8	*Enterobacter*	Enterobac C
9	*Enterococcus*	Enteroc A
10	*Enterococcus*	Enteroc A
11	*Enterococcus*	Enteroc B
12	*Enterococcus*	Enteroc B
13	*Enterococcus*	Enteroc C
14	*Enterococcus*	Enteroc C
15	*Enterococcus*	Enteroc D
16	*Enterococcus*	Enteroc E
17	*Escherichia*	Esch A
18	*Escherichia*	Esch A
19	*Escherichia*	Esch B
20	*Escherichia*	Esch B
21	*Escherichia*	Esch C
22	*Escherichia*	Esch C
23	*Escherichia*	Esch D
24	*Escherichia*	Esch D
25	*Escherichia*	Esch E
26	*Escherichia*	Esch E
27	*Klebsiella*	Kleb A
28	*Klebsiella*	Kleb A
29	*Klebsiella*	Kleb B
30	*Klebsiella*	Kleb C
31	*Klebsiella*	Kleb C

**Fig. 1:**
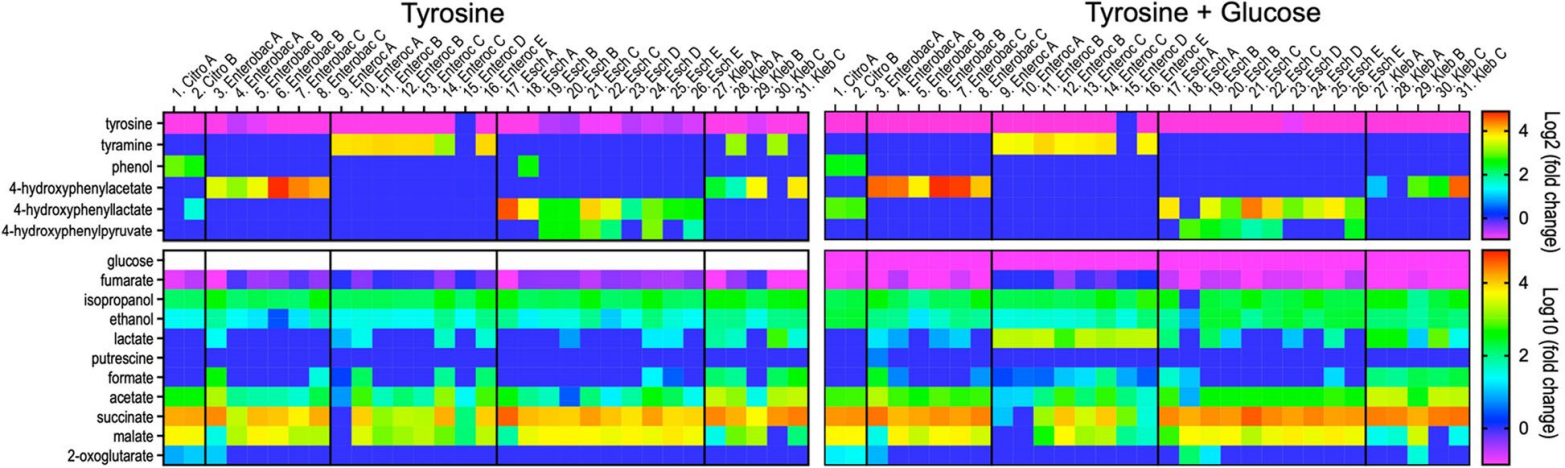
Metabolite production and utilization of bacterial isolates cultured anaerobically using a simple base medium supplemented with tyrosine (left panel) or tyrosine and glucose (right panel). Fold changes of relative concentrations of metabolites in bacterial media to the control medium without bacteria were calculated. Negative values of log2 (fold change) or log10 (fold change) represent substrate utilization (e.g., glucose, fumarate, and tyrosine), whereas positive values represent metabolite production (e.g., tyramine, succinate). Bacterial information can be found in Table 1

We subsequently evaluated the tyrosine degradation capabilities of these bacterial isolates under anaerobic conditions using a simple medium base supplemented with tyrosine or tyrosine and glucose. Metabolites were detected and their relative concentrations were quantitated using ^1^H NMR spectroscopy. While all isolates, except for Enteroc D (no. 15), consumed tyrosine, *Citrobacter* produced phenol as a main product of tyrosine, whereas *Enterobacter* and *Klebsiella* produced 4-hydroxyphenylacetate, *Escherichia* produced 4-hydroxyphenyllactate and 4-hydroxyphenylpyruvate, and *Enterococcus* and two *Klebsiella* isolates produced tyramine (Fig. 1 top panel and Fig. S1 representative NMR spectra). These observations suggested that *Enterococcus* and some *Klebsiella* isolates could partially contribute to the elevated fecal tyramine levels observed previously in patients post-RYGB [8]. Furthermore, we observed that *Enterococcus* utilized less fumarate compared to *Enterobacteriaceae* bacteria (Fig. 1 bottom panel). *Enterococcus* was a strong lactate producer when cultured in the glucose-supplemented medium; *Enterobacteriaceae* bacteria produced higher concentrations of succinate and malate. The metabolic activities of these isolates under aerobic conditions are described in the Supplementary information (Fig. S2).

### Tyramine-induced cell death and DNA damage in HCT 116 cells

We previously showed that RYGB surgery-associated *Enterobacteriaceae* family did not colonize in the gut of non-operated rats following RYGB fecal microbiota transplantation, nor induce any metabolic changes in fecal and urinary metabolome [47]. Furthermore, our pilot experiment showed that the tyramine-producing *Klebsiella* isolates did not colonize the gut of the wild-type and *Apc*^*Min/*+^ mice. Hence, we directly explored the impact of RYGB surgery-associated fecal metabolites on colonic health, focusing on tyramine as fecal tyramine has been shown to be associated with both RYGB surgery and IBD [8, 19, 20].

**Fig. 2:**
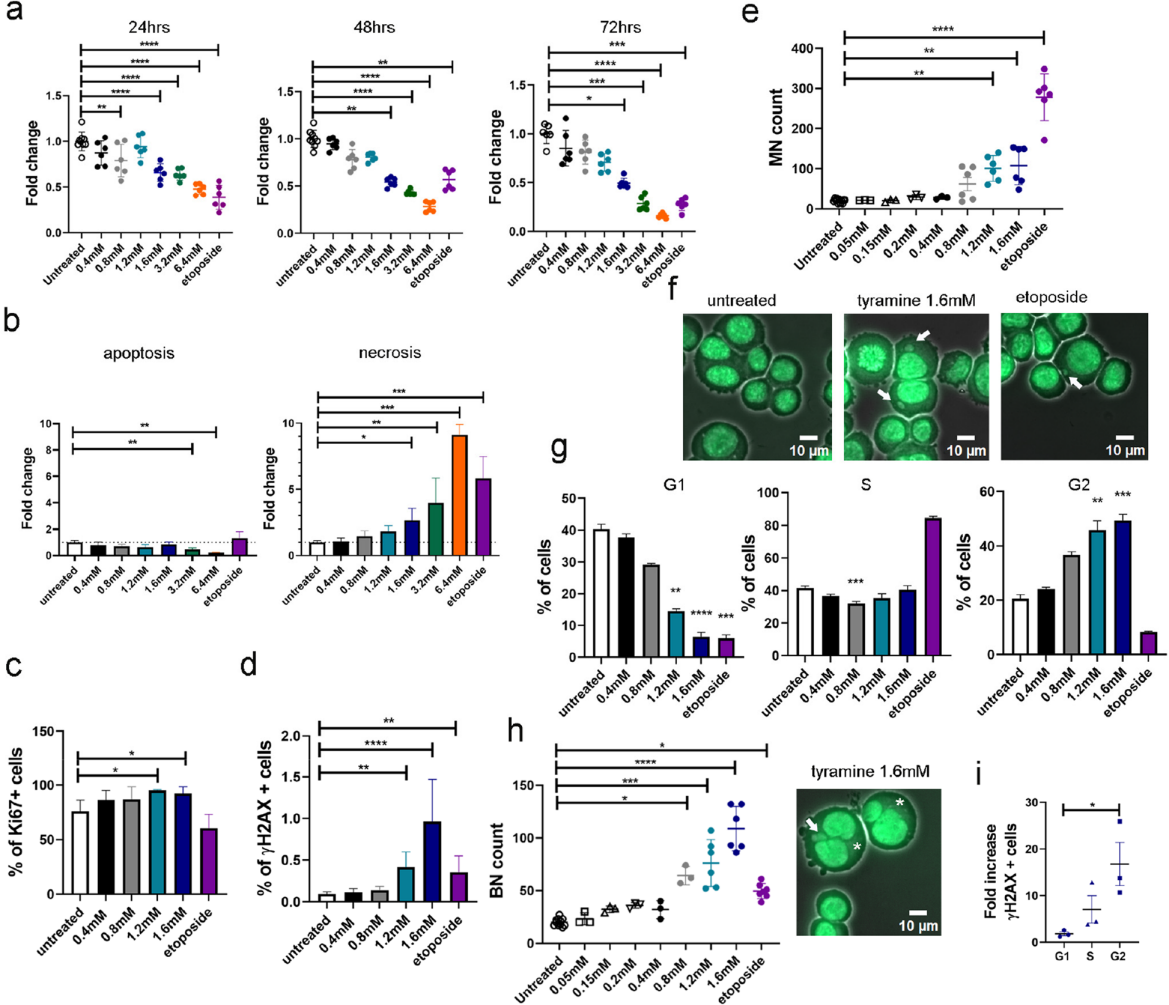
Tyramine reduced cell viability mainly due to necrosis and increased the number of micronucleated and binucleated cells. A range of concentrations of tyramine was added in human colorectal cancer HCT 116 cells over a series of time points. Untreated cells and etoposide served as negative and positive control respectively for cytotoxicity and genotoxicity. **a** Fold change of cell viability measured as fluorescence intensity of alamarBlue reagent added to HCT 116 cells treated with various concentrations of tyramine for 24, 48, and 72 h (*n* = 6–9 per group) over untreated control cells. One-Way ANOVA with Dunnett’s multiple comparisons test was applied for 24 h. Kruskal–Wallis test with Dunn’s multiple comparisons test for 48 and 72 h. Results were pooled from 3 independent experiments per time point. **b** Fold change of apoptotic and necrotic cells acquired with flow cytometry using Annexin V and Helix NP Blue DNA dye after 24 h of treatment with tyramine relative to untreated control. Data from two independent experiments were pooled (*n* = 6 per group). Kruskal–Wallis test with Dunn’s multiple comparisons test. **c** Percentage of Ki67-positive cells after treatment with varying concentrations of tyramine for 24 h. Data from two independent experiments were combined (*n* = 8 per group). Kruskal–Wallis test with Dunn’s multiple comparisons test. **d** Percentage of phosphorylated H2AX (γH2AX) positive cells treated with tyramine for 24 h. Data from two independent experiments were combined (*n* = 8 per group). Kruskal–Wallis test with Dunn’s multiple comparisons test. **e** Number of micronuclei (MN) per 2000 cells after 48 h treatment. Slides were scored blindly. Kruskal–Wallis test with Dunn’s multiple comparisons test. **f** Representative fluorescence microscopy images from MN genotoxicity assay in untreated cells, and cells treated with tyramine 1.6 mM and etoposide. Arrowheads show the presence of MN. Scale bar 10 µm. **g** Frequency of cells at different stages of the cell cycle, namely G1, S, and G2/M after 24 h treatment. Kruskal–Wallis test with Dunn’s multiple comparisons test. *N* = 6 per group apart from the etoposide group (*n* = 3). **h** Count of cells with binuclei (BN) per 2000 cells and an image of BN in 1.6 mM tyramine on the right. Scale bar 10 µm. Kruskal–Wallis test with Dunn’s multiple comparisons test. For **e** and **h** data from three independent experiments were pooled (*n* = 6 for untreated, 1.2 mM tyramine, 1.6 mM tyramine and etoposide, *n* = 3 for 0.05 mM, 0.15 mM, 0.2 mM, 0.4 mM, and 0.8 mM tyramine). Arrowheads show the presence of MN and asterisks of BN cells. Three slides were scored per treatment. **i** Fold change of γH2AX positive cells at different stages of the cell cycle after 24 h treatment with 1.6 mM tyramine over untreated cells. *X*-axis indicated cell cycle stages: circle, G1 phase; triangle, S phase; rectangular, G2 phase. Data representative from two independent experiments with 3 technical replicates per experiment. *X*-axis indicated cell treatment groups in **a**–**e**, **g**, and **h**, and the color coding used throughout these graphs is: white, untreated; black, 0.4 mM tyramine; grey, 0.8 mM tyramine; petrol, 1.2 mM tyramine; dark blue, 1.6 mM tyramine; green, 3.2 mM tyramine; orange, 6.4 mM tyramine; purple, etoposide. Kruskal–Wallis test with Dunn’s multiple comparisons test in **a**–**e** and **g**–**i**. One-Way ANOVA with Dunnett’s multiple comparisons test was applied for **a** 24 h. Data is shown as means ± SD for all graphs. *P < 0.05; **P < 0.01, ***P < 0.001; ****P < 0.0001

To examine the cytotoxic effects of tyramine, HCT 116 cells were treated with 0, 0.4, 0.8, 1.2, 1.6, 3.2, or 6.4 mM of tyramine for 24, 48, or 72 h. We observed that tyramine was cytotoxic at a concentration of 0.8 mM at 24 h, and at a concentration of 1.6 mM or higher the cytotoxic effect was persistent up to 72 h (Fig. 2a). A drop in cell viability at 1.2 mM at 48 and 72 h was noted without reaching statistical significance. Cells treated with low concentrations (0.05, 0.15, and 0.2 mM) of tyramine displayed no cytotoxicity (Fig. S3). We further evaluated if tyramine-induced cytotoxicity was contributed by cell apoptosis and/or necrosis by staining live cells with Annexin V and Helix NP Blue dye (Fig. S4a). The frequency of necrotic cells increased in response to tyramine treatment in a dose-dependent manner (Fig. 2b). A significant decrease in apoptotic cell numbers compared to untreated cells was observed at the treatment concentrations of 3.2 and 6.4 mM (Fig. 2b). In addition, the nuclei without plasma membrane significantly increased with tyramine treatment at a concentration of 1.2 mM or above compared to the untreated control (Fig. S4b).

Increased cell proliferation and DNA instability are known risk factors for tumorigenesis [48]. We, therefore, investigated whether tyramine affects cell proliferation and induces DNA damage. The percentage of Ki67 positive cells was significantly higher 24 h after the addition of tyramine at 1.2 mM (*P* = 0.0131) and 1.6 mM (*P* = 0.0323) concentrations compared to the untreated controls (Fig. 2c; Fig. S4c). At these treatment concentrations, a significantly higher frequency of phosphorylated histone H2AX cells, forming γH2AX, was observed in comparison to untreated controls (*P* = 0.001 for 1.2 mM tyramine and *P* < 0.0001 for 1.6 mM tyramine) (Fig. 2d). This observation indicates a cellular response to the induction of DNA double-strand breaks [49]. Additionally, we assessed tyramine-induced DNA damage using the MN assay. MN are small extra-nuclear bodies that contain acentric chromosome fragments and/or whole chromosomes. They occur as a consequence of extensive DNA damage, such as double-strand breaks and/or deficiencies in chromosome segregation during anaphase [50]. Concentrations of tyramine at 1.2 mM and 1.6 mM induced the formation of MN (Fig. 2e, f), indicating increased DNA damage and chromosomal aberrations. We further showed that unlike the positive control etoposide that targets directly topoisomerase II generating DNA strand breaks [51], tyramine at 1.6 mM did not induce a significant genotoxic effect within 6 h of treatment (Fig. S4d).

In addition to increased cell proliferation and DNA damage, tyramine at 1.2 mM and 1.6 mM induced cell cycle arrest at the G2/M phase (Fig. 2g) and an elevated number of binucleated cells (Fig. 2h). A significantly higher frequency of γH2AX positive cells following tyramine treatment was found in G2 phase (*P* = 0.0338), suggesting the activation of DNA repair mechanisms at this phage (Fig. 2i). Metabolic profiles of cell media from tyramine-treated cells (0.8, 1.2, and 1.6 mM) showed separated clusters in the scores plot of principal component analysis (PCA) and concentrations of lactate, a byproduct of glucose metabolism, reduced as the tyramine treatment concentrations increased, suggesting that tyramine-induced cell cycle arrest affects cellular metabolism (Fig. S5).

Overall, we showed that tyramine treatment resulted in cell necrosis, and promoted cell proliferation and DNA damage, with an enhanced DNA repair at the G2 phase of the cell cycle. These effects are expected to compromise genomic stability and increase the risk of colonic tumorigenesis.

### Tyramine affected DNA organization and oxidative respiration pathways in HCT 116 cells

**Fig. 3:**
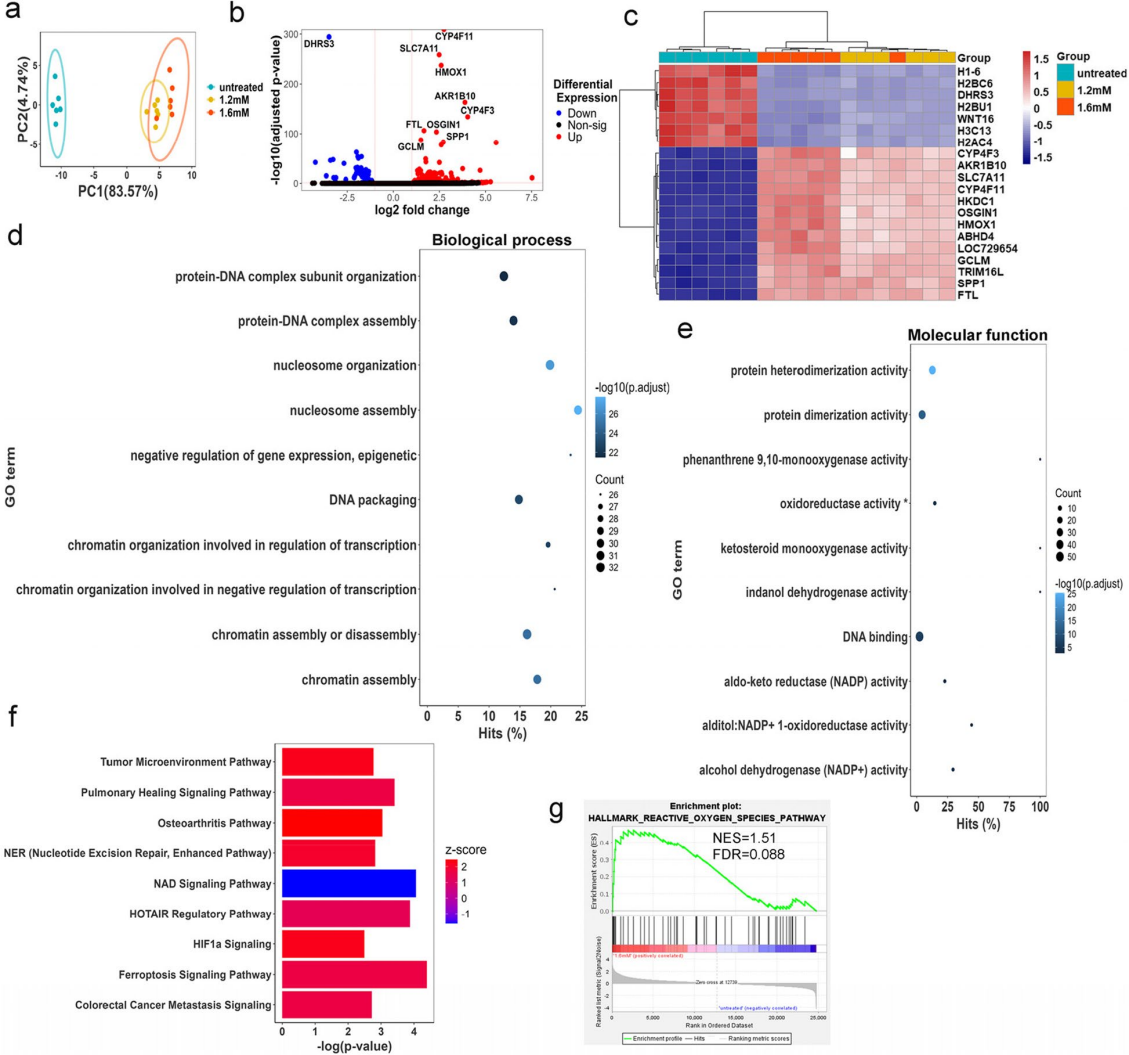
Tyramine alters gene expression profiles and pathways involved in chromatin conformation, oxidative reductive reactions, and colorectal cancer signaling. RNAseq analysis using DESeq2 on cells treated with 1.2 mM or 1.6 mM tyramine for 24 h or left untreated (*n* = 6 per group). **a** Principal components analysis (PCA) plot showing the clustering of variance stabilizing transformation (VST) RNAseq data. **b** Volcano plot of genes enriched or reduced in 1.6 mM tyramine treatment versus control. The dotted vertical lines enclose the minimum fold change for the most significant genes. A cut-off of > 2 absolute value was used for fold change and < 0.05 for FDR-corrected p-value to assign a gene as differentially expressed. The 10 most differentially expressed genes are shown in the plot. **c** Heatmap of the top 20 genes using *z*-scores of genes that are differentially expressed between 1.6 mM or 1.2 mM tyramine treatment group versus control. Each column in the heatmap is an individual sample. **d** Plot of top 10 over-represented GO terms to visualize the biological processes and** e** molecular functions altered in 1.6 mM tyramine-treated cells compared to controls. Hits reflect the proportion of differentially expressed genes in a given pathway. The number of altered genes is indicated by the size of the circle area, and the circle color represents the ranges of the corrected *P* value. FDR correction < 0.05 has been applied to the depicted GO terms.** f** IPA pathways that were significantly upregulated (*z*-score > 0) or downregulated (*z*-score < 0) in tyramine 1.6 mM versus control. Pathways with absolute *z*-score > 1 are depicted. **g** Gene set enrichment plot demonstrating the correlation of gene sets involved in oxygen species pathway with 1.6 mM tyramine. Gene permutation was used. FDR correction < 0.05 has been applied. NES, normalized enrichment score. Abbreviations: ABHD4, abhydrolase domain containing 4, N-acyl phospholipase B; AKR1B10, aldo–keto reductase family 1 member B10; CYP4F3, cytochrome P450 family 4 subfamily F member 3; CYP4F11, cytochrome P450 family 4 subfamily F member 11; DHRS3, dehydrogenase/reductase 3; FTL, ferritin light chain; GCLM, glutamate-cysteine ligase modifier subunit; HKDC1, hexokinase domain containing 1; HMOX1, heme oxygenase 1; H1-6, H1.6 linker histone, cluster member; H2AC4, H2A clustered histone 4; H2BC6, H2B clustered histone 6; H2BU1, H2B.U histone 1; H3C13, H3 clustered histone 13; LOC729654, inositol polyphosphate multikinase pseudogene; OSGIN1, oxidative stress induced growth inhibitor 1; SLC7A11, solute carrier family 7 member 11; SPP1, secreted phosphoprotein 1; TRIM16L, tripartite motif containing 16 like; WNT16, Wnt family member 16. * denotes oxidoreductase activity, acting on paired donors, with incorporation or reduction of molecular oxygen, NAD(P)H as one donor, and incorporation of one atom of oxygen

To investigate the mechanism of action of tyramine, we performed deep RNA sequencing on untreated control cells and cells treated with 1.2 mM or 1.6 mM of tyramine for 24 h, which induced significant cytotoxicity and genotoxicity in HCT 116 cells. The PCA scores plot of the gene expression profiles displayed a clear separation between control and tyramine-treated cells (Fig. 3a). The two tyramine doses induced a similar pattern of cellular responses to a lesser extent from the low dose group. A total of 524 and 241 differentially expressed genes in 1.6 mM and 1.2 mM tyramine-treated cells versus untreated controls were found (Fig. 3b and Fig. S6a). Heatmaps displayed differentially expressed gene sets between tyramine and control groups (Fig. 3c and Fig. S6b). Among the most significantly altered genes were histones (e.g., H1-6, H2AC4, H2BC6, H2BU1, H3C13), which were downregulated, whereas genes involved in oxidation/reduction reactions and oxidative stress responses (e.g., AKR1B10, CYP4F3, CYP4F11, OSGIN1) were upregulated in both tyramine-treated groups (Fig. 3c). Short-chain dehydrogenase/reductase 3 (DHRS3) was significantly down-regulated in tyramine-treated groups. Reduced expression of DHRS3 has been previously shown in gastric cancer, and its ectopic expression was found to inhibit cell proliferation and migration [52]. Expression of genes involved in glucose metabolism such as HKDC1 [53] and amino acid metabolism such as SLC7A1 [54], were elevated, suggesting an impact of tyramine on cell energy metabolism.

Pathway analysis of gene ontology (GO) terms referring to biological processes revealed that chromatin organization and assembly networks were significantly reduced in the 1.6 mM tyramine-treated group with an impact on the regulation of transcription and epigenetic control of gene expression (Fig. 3d). Chromosomal instability is one of the major routes that leads to CRC and is characterized by structural chromosomal abnormalities, aneuploidy, loss of heterozygosity, and chromosomal rearrangements [55]. Therefore, our observation of compromised chromatin assembly and elevated MN frequency strongly indicates signs of chromosomal instability in the presence of tyramine. Furthermore, oxidation and reduction reactions involving NADP/NAD were markedly reduced, suggesting the involvement of mitochondria function (Fig. 3e and Fig. S6c). Additionally, IPA analysis consistently showed increased oxidative stress responses and DNA damage repair following tyramine treatment, together with down-regulation of the NAD signaling pathway (Fig. 3f). Moreover, processes related to colorectal cancer metastasis signaling and tumor microenvironment were enhanced. Ferroptotic cell death, which is iron-dependent and results from the accumulation of lipid reactive oxygen species (ROS) [56], was higher in the tyramine group. In further support of the role of tyramine in oxidative damage, Gene Set Enrichment Analysis (GSEA) showed an overrepresentation of gene sets in reactive oxygen species (Fig. 3g and Fig. S6d). Analogous findings of gene pathway analysis were also found in the 1.2 mM tyramine group (Fig. S7). The RNA sequencing data analyses collectively indicate that tyramine impacted DNA structure and resulted in oxidative stress, which may be responsible for its genotoxic and cytotoxic effects.

### Tyramine increased CRC risk in WT mice

**Fig. 4:**
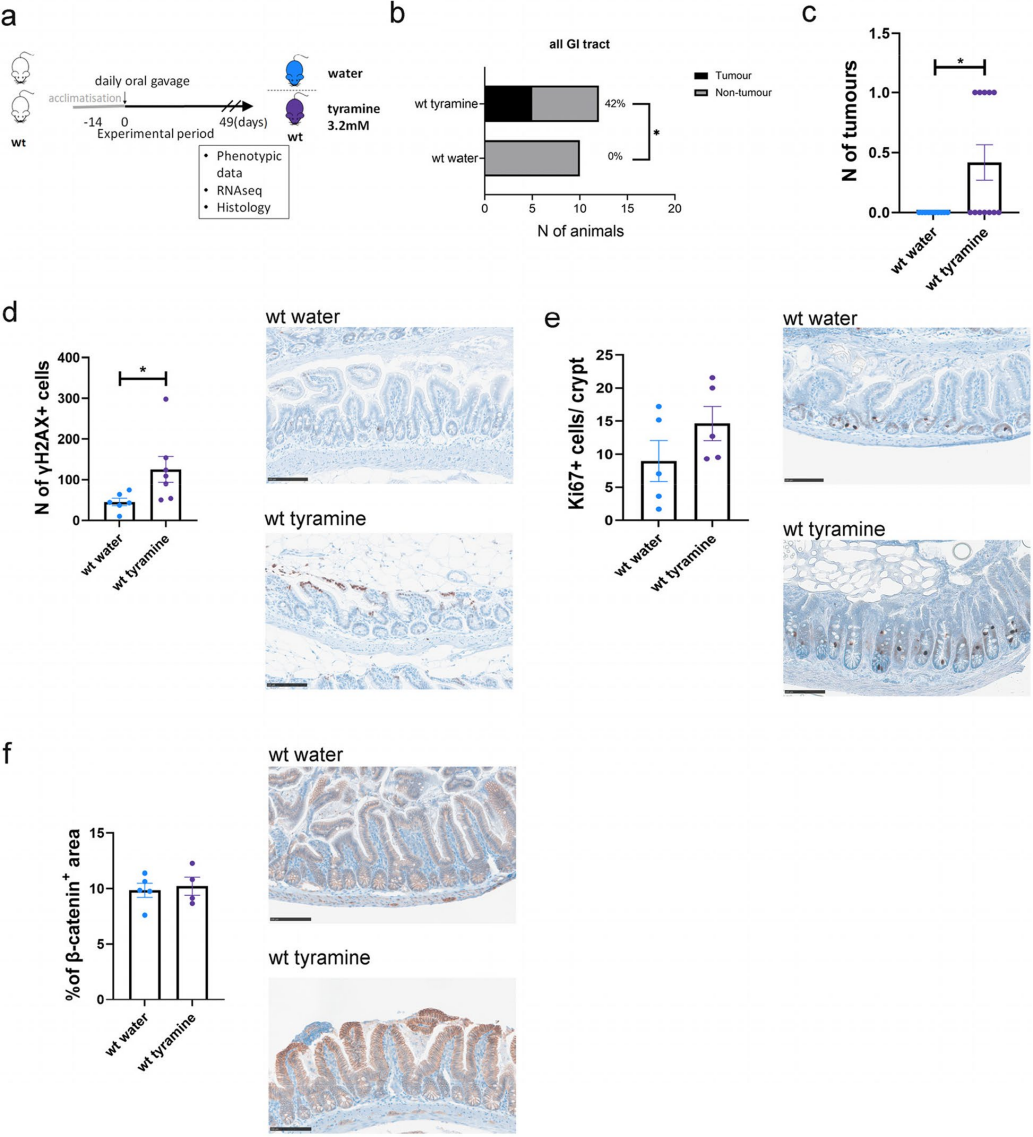
Tyramine increases the number of cells in the intestine of WT mice exhibiting signs of DNA damage. WT littermate controls received daily doses of 200 µl of 3.2 mM tyramine (*n* = 12) or water (*n* = 10) via oral gavage for a period of 49 days. Treatment started at 6 weeks of age and concluded at 13 weeks of age. **a** Schematic of the study design. **b** Contingency table showing the number of animals with tumors. The percentages of animals having tumors per group are also depicted. **c** Total tumor numbers of the small and large intestines were counted in tyramine-treated and untreated WT mice. **d** Number of γH2AX positive cells per field of view and representative immunohistochemistry images in the ileum. Three separate fields of view were taken for each tissue sample. **e** Number of Ki67 + cells per crypt in the ileum and representative immunohistochemistry images. A total of 60 crypts per slide was counted. Three slides were acquired for each tissue sample. **f** Quantification of β-catenin positive area in the ileum and representative immunohistochemistry images. Light blue, water-treated WT mice; purple, tyramine-treated WT mice. Scale bar 100 µm. Data are shown as means ± SEM. Fisher's exact test in **b** and Mann–Whitney test in **c**–**f**. *P < 0.05

Following up on our observations of tyramine-induced cell proliferation and genotoxicity in vitro, we investigated if tyramine increases CRC risk in WT mice, which received 200 µL of 3.2 mM tyramine solution or water daily for 49 days (Fig. 4a). The dosage was determined based on dietary intake of foods that contain high amounts of tyramine in humans [57–59] and adjusted to the average weight of mice. A striking finding was that 42% of tyramine-treated WT mice developed one intestinal tumor each (Fig. 4b, c). This observation is in line with a significantly enhanced number of γH2AX + cells in the ileum (*P* = 0.014) of tyramine-treated WT mice, suggesting increased DNA damage compared to controls (Fig. 4d). In addition, a higher number of Ki67 + cells were observed in the ileum of the tyramine group compared to the control but not reaching statistical significance (Fig. 4e). The levels of β-catenin levels were comparable between the two groups (Fig. 4f). Our data collectively suggest that tyramine increased cell proliferation and significantly promoted DNA damage and intestinal tumorigenesis in WT mice.

### Tyramine impacted the cell cycle and epithelial barrier function pathways in the ileum and colon of WT mice

**Fig. 5:**
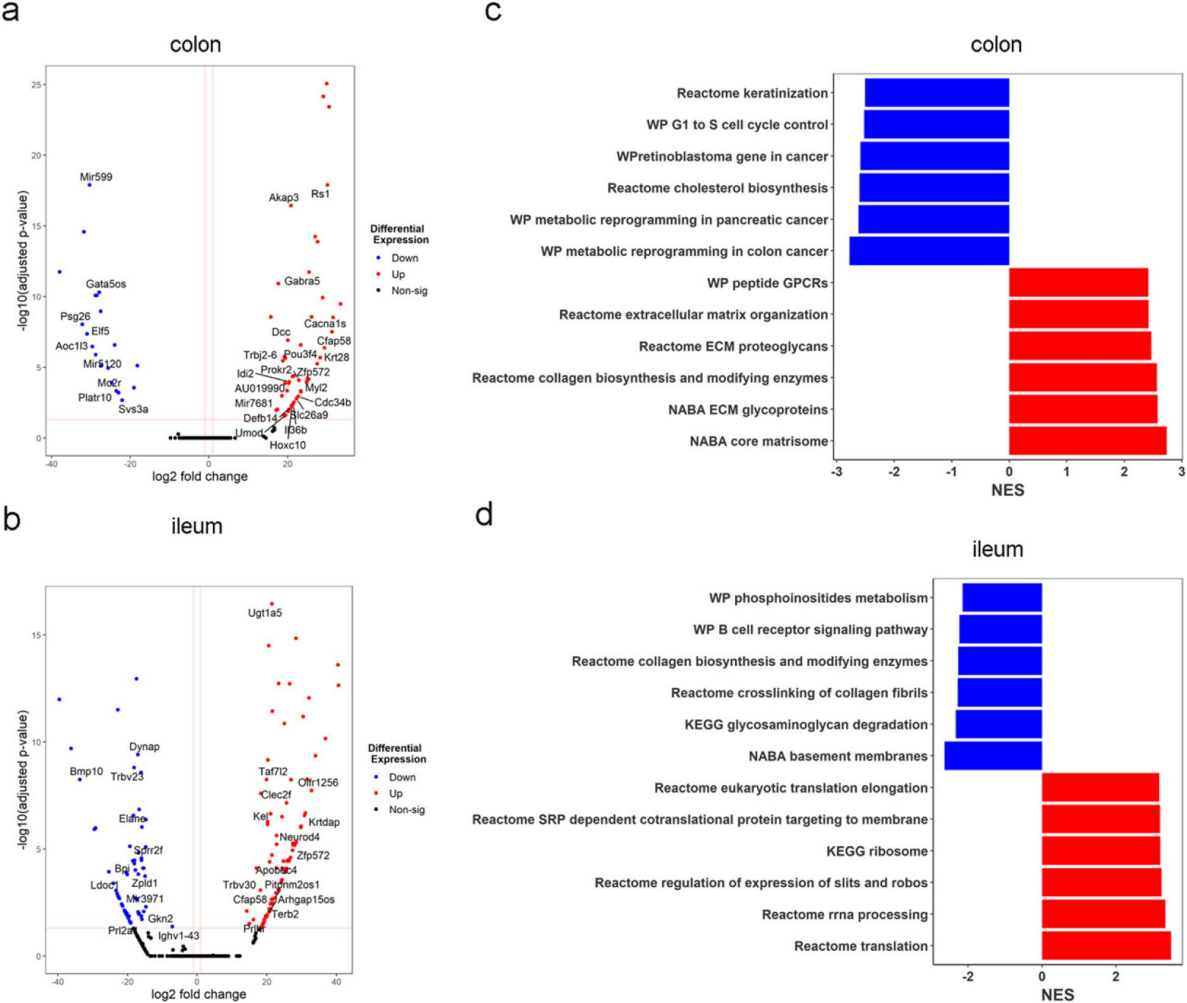
Tyramine alters the expression profiles of genes involved in epithelial barrier function in WT mice. RNAseq analysis using DESeq2 on colon and ileum tissue of WT mice treated with tyramine 3.2 mM or water for 7 weeks (*n* = 5 for colon tyramine-treated WT mice, *n* = 4 for colon water-treated WT mice, and *n* = 9 for ileum tyramine-treated WT mice, *n* = 8 for ileum water-treated WT mice). Volcano plot of genes enriched or reduced in tyramine treatment versus control in **a** colon and **b** ileum. The dotted vertical lines enclose the minimum fold change for the most significant genes. A cut-off of > 2 absolute value was used for fold change and < 0.05 for FDR-corrected p-value to assign a gene as differentially expressed. Differentially expressed genes are shown on the plots. Abbreviations of some interesting genes: *Cdc34b*, cell division cycle 34B; *Defb14*, defensin beta 14; *Clec2f*, C-type lectin domain family 2, member f.** c** GSEA of the top 6 most significant downregulated and upregulated pathways in colon and **d** ileum. FDR correction was applied. NES, normalized enrichment score

We found a significant upregulation of the cell division cycle gene (*Cdc34b*) expression in the colon of tyramine-treated WT mice compared to water-treated mice (Fig. 5a). Moreover, the expression levels of genes related to epithelial barrier function, including defensins and C-type lectins (*Defb14* and *Clec2f*) were elevated in the colon and ileum, respectively, in the tyramine-treated WT mice compared to water-treated WT mice (Fig. 5b). Similar to tyramine-treated *Apc*^*Min/*+^ mice, pathways related to extracellular matrix components were overrepresented in the colon of the tyramine-treated WT mice, whereas cell cycle control from G1 to S phase was diminished, suggesting reduced control in cell proliferation (Fig. 5c). Protein translation and synthesis pathways (i.e., reactome eukaryotic translation elongation, Reactome SRP dependent cotranslational protein targeting to membrane, KEGG ribosome, Reactome regulation of expression of Slits and Robos, Reactome rRNA processing, Reactome translation) were upregulated in the tyramine-administered group in the ileum, suggesting increased cell division (Fig. 5d). These observations are consistent with higher levels of Ki67 + cells in tyramine-treated WT mice and tyramine-treated HCT 116 cells in contrast to their untreated controls. Collectively, our data suggest that tyramine reduced the control of cell proliferation and cell cycle, contributing to tumor initiation in WT mice.

### Tyramine facilitated tumorigenesis and increased cell proliferation in *Apc*^*Min/*+^ mice

**Fig. 6:**
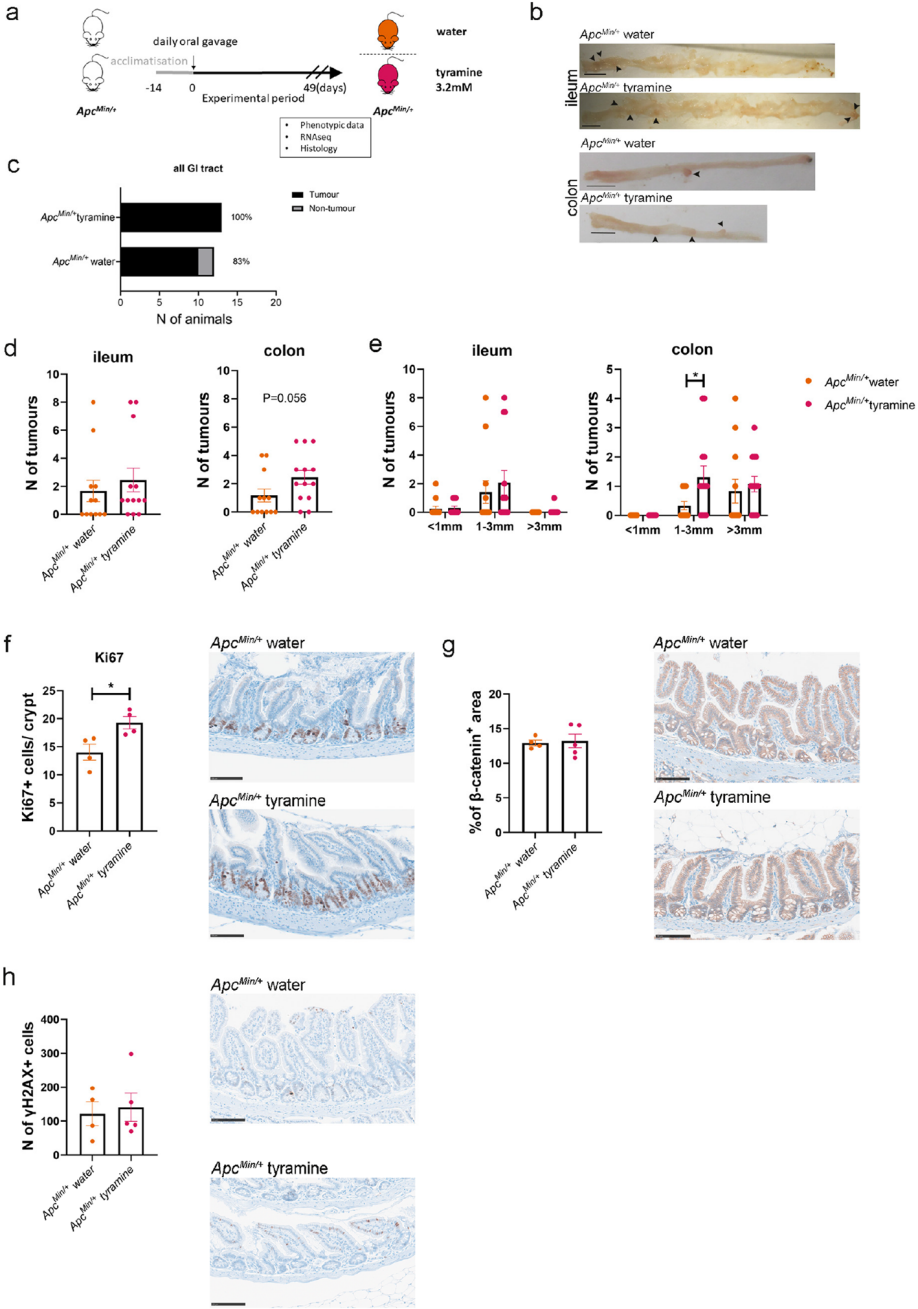
Tyramine promotes CRC development in *Apc*^*Min/*+^ mice increasing colonic tumor size and enhancing epithelial cell proliferation. *Apc*^*Min/*+^ mice received daily doses of 200 µl of 3.2 mM tyramine (*n* = 13) or water (*n* = 12) via oral gavage for a period of 49 days. Treatment started at 6 weeks of age and concluded at 13 weeks of age. **a** Schematic of the study design. **b** Representative macroscopic images of ileum and colon in *Apc*^*Min/+*^ mice administered water or tyramine. Scale bars, 1 cm. **c** The number of animals with or without tumors. The percentages of animals having tumors per group are also depicted. **d** Tumor load in ileum and colon at study endpoint. **e** Tumor numbers based on tumor size in the ileum and colon. Tumors smaller than 1 mm, between 1 and 3 mm, and greater than 3 mm were counted separately. **f** Number of Ki67 + cells per crypt in the ileum and representative immunohistochemistry images. A total of 60 crypts per slide was counted. 3 slides were acquired for each tissue sample. Scale bar 100 µm. **g** Quantification of β-catenin positive area and representative immunohistochemistry images. Scale bar 100 µm. **h** Number of γH2AX positive cells per field of view and representative immunohistochemistry images in the ileum. Three separate fields of view were taken for each tissue sample. Scale bar 100 µm. Orange, water-treated *Apc*^*Min/+*^ mice; pink, tyramine-treated *Apc*^*Min/+*^ mice. Data are shown as means ± SEM. Fisher’s exact test in **c**. Multiple Mann–Whitney tests followed by FDR approach for multiple comparisons in **e**, and Mann–Whitney test in **d**, and **f**–**h**. *P < 0.05

We next investigated if tyramine facilitates tumorigenesis using the *Apc*^*Min/*+^ mouse model. *Apc*^*Min/*+^ mice were administered 200 µL of 3.2 mM tyramine solution or water for a period of 49 days (Fig. 6a). Macroscopic evaluation showed that *Apc*^*Min/*+^ mice receiving tyramine tended to develop a higher number of tumors, especially in the colon (Fig. 6b). The number of mice and the percentages of mice with tumors were consistently higher in the tyramine-treated *Apc*^*Min/*+^ group compared with the water-treated *Apc*^*Min/*+^ group across the GI tract (Fig. 6c and Fig. S8a). Despite that the total number of tumors did not reach statistically significant differences between tyramine-treated and water-treated *Apc*^*Min/*+^ mice (Fig. 6d, Fig. S8b–d), tyramine showed an effect on the tumor size. The diameters of intestinal adenomas in mice were measured and allocated to one of three categories: diameter < 1 mm, 1–3 mm, and > 3 mm. The number of colonic tumors with a diameter of 1–3 mm was significantly higher in tyramine-treated *Apc*^*Min/*+^ mice compared with untreated *Apc*^*Min/*+^ mice (Fig. 8e, P = 0.038; Fig. S8e). Furthermore, appearance scoring demonstrated that tyramine administration tended to exacerbate the pathology in *Apc*^*Min/*+^ mice (Fig. S8f). The total number of tumors displayed a positive correlation with spleen weight (*r* = 0.79, *P* = 2.7 × 10^−11^; Fig. S8g).

In addition to increases in colonic tumor size, tyramine treatment resulted in a statistically higher number of Ki67 + cells in *Apc*^*Min/+*^ mice (Fig. 6f, *P* = 0.028). In contrast, β-catenin levels were similar (Fig. 6g). These observations indicate that tyramine increases cell proliferation without affecting the Wnt/β-catenin signaling pathway. Furthermore, a higher number of γH2AX + cells was noted in tyramine-treated *Apc*^*Min/*+^ compared to the untreated group but did not reach statistical significance (Fig. 6h). These data collectively showed that tyramine facilitated intestinal tumorigenesis in *Apc*^*Min/*+^ mice.

### Tyramine impacted gene pathways involved in inflammation and extracellular matrix in the tumor-surrounding ileum and colon tissue of *Apc*^*Min/*+^ mice

**Fig. 7:**
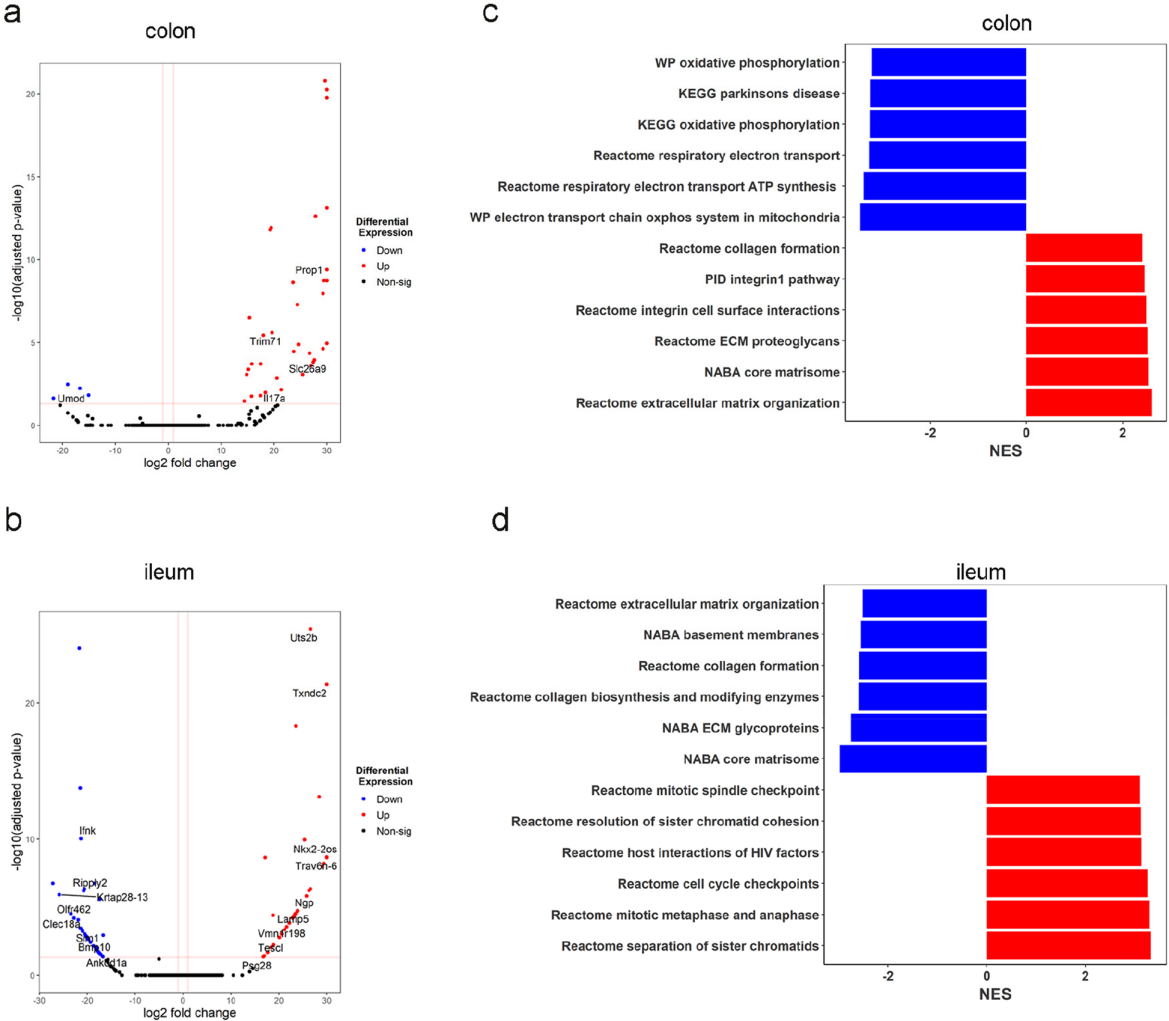
Tyramine promotes an inflammatory environment in surrounding healthy gut tissue instigating tumor initiation in *Apc*^*Min/*+^ mice. RNAseq analysis using DESeq2 on colon and ileum tissue of *Apc*^*Min/*+^ mice treated with tyramine 3.2 mM or water (*n* = 6 for colon tyramine-treated *Apc*^*Min/*+^ mice, *n* = 5 for colon water-treated *Apc*^*Min/*+^ mice, and *n* = 10 per each group for ileum). Volcano plot of genes enriched or reduced in tyramine treatment versus control in **a** colon and **b** ileum. The dotted vertical lines enclose the minimum fold change for the most significant genes. A cut-off of > 2 absolute value was used for fold change and < 0.05 for FDR-corrected p-value to assign a gene as differentially expressed. Differentially expressed genes are shown in the plot. Abbreviations of some interesting genes: *IL17a*, interleukin-17A; *Ngp*, neutrophilic granule protein. **c** GSEA of the top 6 most significant downregulated and upregulated pathways in the colon and **d** ileum. FDR correction was applied. NES, normalized enrichment score

Tyramine binds to a family of G-protein-coupled receptors, termed trace amine-associated receptors (TAARs), which are expressed in the intestine [23]. Tyramine binds to TAAR1 expressed in various immune cell subsets including macrophages, natural killer (NK) cells, neutrophils, T and B cells, and mediates changes in cytokine secretion and chemotaxis [60]. Specifically, tyramine binding to TAAR1 in macrophages increased inflammatory gene expression [61]*.* To gain further insight into the function of tyramine in tumorigenesis in vivo, we performed transcriptomic analysis of tumor-surrounding colon and ileum tissues of *Apc*^*Min/*+^ mice. Tyramine treatment upregulated colonic tissue expression of *IL-17A* gene compared to untreated *Apc*^*Min/*+^ mice (Fig. 7a). IL-17A is a pro-inflammatory cytokine that drives intestinal tumorigenesis [62, 63] and it is required for tumor formation in *Apc*^*Min/*+^ mice [64]. In addition, we observed higher expression levels of neutrophilic granule protein (*Ngp*) gene in the ileum tissue of tyramine-treated *Apc*^*Min/*+^ mice in contrast to untreated *Apc*^*Min/*+^ mice (Fig. 7b). This observation is consistent with the immunohistochemistry analysis of the ileum tissue, which showed a higher number of Ly6G + cells (neutrophils) in *Apc*^*Min/*+^ mice following tyramine treatment compared to untreated *Apc*^*Min/*+^ mice (Fig. S9a).

GSEA pathway analysis showed enrichment of genes involved in extracellular matrix organization and decreases in mitochondrial activities in the colon tissue following tyramine treatment (Fig. 7c). In contrast, extracellular matrix organization pathway was downregulated in the ileum, together with collagen biosynthesis pathways in the tyramine-treated *Apc*^*Min/*+^ mice compared to untreated ones (Fig. 7d), which may indicate changes in gut epithelial barrier. Although both the ileum and colon tissue of *Apc*^*Min/*+^ mice showed upregulated expression levels of genes involved in inflammatory responses to tyramine, these different genes involved in two tissue types could be attributed to their physiological differences and possibly differential binding of tyramine to its relevant receptors across the intestine [23].

### Tyramine promoted a tumorigenic immune response in the tumor microenvironment

We next sought to characterize the immune cell subsets in the tumors from tyramine or water-treated *Apc*^*Min/*+^ mice using flow cytometry (Fig. S10a–b). Owing to the large variation in tumor numbers and size from the *Apc*^*Min/*+^ mice, we were able to collect enough colonic tumors from 3 control and 4 tyramine-treated *Apc*^*Min/*+^ mice for this analysis. We observed similar frequencies on lymphoid, innate lymphoid, and myeloid cells in colonic tumor-infiltrating lymphocytes (TIL) (Fig. S9b, Fig. S11, and Fig. S12). In contrast, the frequency of NK cells was lower in *Apc*^*Min/*+^ mice treated with tyramine compared to those treated with water (Fig. S9b), which was accompanied by a reduction in the mean fluorescence intensity (MFI) of NKG2D in NK cells (Fig. S9c). NK cells, in particular NKG2D-expressing NK cells, are important players in the anti-tumor response due to their ability to recognize and eliminate tumor cells without the need for antigen-specific receptors [65, 66]. Our observation of a reduction in NKG2D-expressing NK cells suggested that tyramine may compromise an effective anti-tumor response at the early stage of tumorigenesis. On the other hand, polymorphonuclear-myeloid-derived suppressor cells (PMN-MDSCs) displayed an increased trend in their proportion in the tyramine-treated group (Fig. S9b). PMN-MDSCs have several functions but are generally thought to suppress the anti-tumor response, thereby promoting tumor growth [67, 68]. Furthermore, MFI of the inhibitory marker PD-1 showed a higher frequency in CD8 + T cells but did not reach statistical significance (Fig. S9d), suggesting previous activation and potential exhaustion of cytotoxic CD8 + T cells. Tumor-specific CD8 + T cells become exhausted due to persistent stimulation impairing their effector function against tumor progression [69]. In the tumors, gene pathways including extracellular matrix glycoproteins and basement membranes (BM) were down-regulated by tyramine treatment, which may indicate an epithelial-mesenchymal transition (EMT) in the tumors and invasion to the surrounding stroma through BM [70] (Fig. S9e). Our findings suggest that tyramine suppresses an anti-tumor immune response to favor tumor progression in the colonic tumor microenvironment.

## Discussion

We reported genus-specific metabolic patterns in tyrosine metabolism of *Enterobacteriaceae* and *Enterococcaceae* isolates from the feces of patients following RYGB surgery. Consistent with previously reported tyramine production from *E. faecalis* [71], the isolated *Enterococcus* species were strong tyramine producers. The *tdc* clusters in *E. faecalis* encode the tyrosine decarboxylase, which catalyzes the conversion of tyrosine to tyramine [71]. While *Klebsiella* and *Enterobacter* isolates were strong 4-hydroxyphenylacetate producers, two *Klebsiella* isolates were also able to produce tyramine under a glucose-limited anaerobic condition. A *K. pneumonia* strain (NCIMB, 2122) was reported as a medium tyramine former [72], which is in agreement with our observation. 4-Hydroxyphenylacetate is a precursor of *p*-cresol and this conversion is catalyzed by 4-hydroxyphenylacetate decarboxylase (Hpd) [73]. *Coriobacteriaceae, Clostridium* cluster XI, and XIVa have been shown to produce *p*-cresol [73], but to our best knowledge, no strains from *Enterobacteriaceae* family are *p*-cresol-producers. Bacteria, which harbor Hpd but not tyrosine lyase (ThiH), metabolizing tyrosine to *p*-cresol in one step, utilize 4-hydroxyphenylacetate as a substrate to produce *p*-cresol [73]. Our findings showed that *Escherichia* isolates were the main producers of 4-hydroxypenylpyruvate and 4-hydroxypenyllactate. The metabolic conversions from tyrosine to 4-hydroxypenylpyruvate and from 4-hydroxypenylpyruvate to 4-hydroxypenyllactate are catalyzed by tyrosine aminotransferase and hydroxyphenylpyruvate reductase, respectively. Our observations are consistent with previously reported *E. coli* producing 4-hydroxypenylpyruvate and 4-hydroxypenyllactate from tyrosine [73, 74]. Phenol production was found in two strains of *Citrobacter* in our study. It was previously reported that *C. koseri* and *C. freundi* exhibited phenol-producing activities [73, 75] *via* an anaerobic catabolic pathway. An aerobic pathway generating phenol along with pyruvate has been reported in *Enterobacteriaceae* family [76].

These bacterial metabolites from tyrosine are profoundly linked to the reported metabolic changes in fecal water and urine from patients following RYGB surgery. For example, urinary concentrations of 4-hydroxyphenylacetate increased post-surgery, together with 4-cresyl sulfate and 4-cresyl glucuronide, which are host conjugation derivatives from *p*-cresol [8, 13]. While *p*-cresol was not produced in the isolates tested in the current study, these isolates provided the precursor, 4-hydroxyphenylacetate, which could be used in cross-feeding processes within the post-RYGB gut microbial community, allowing the production of *p*-cresol. Higher fecal concentrations of tyramine post-RYGB surgery have also been reported, and RYGB-associated fecal microbiota has been shown to produce tyramine from tyrosine compared to the fecal microbiota of lean donors [8]. In the current study, we further identified the contributors to tyramine production within the RYGB-associated microbiota. Following RYGB, the gut luminal bioavailability of tyrosine for bacterial metabolism may increase, as the surgery reduces pancreatic enzyme production and the absorption of protein-associated amino acids in the small intestine [77]. While dietary tyramine is likely to be primarily absorbed in the small intestine of healthy individuals [78], tyramine absorption post-RYGB remains unclear. Therefore, high fecal tyramine concentrations post-RYGB could be attributed to diet, altered gastrointestinal anatomy, and increased gut bacterial abundances of tyramine-producers.

Since the fecal microbiota associated with RYGB do not colonize the anatomically normal gut [47], we directly examined the impact of its metabolic product, tyramine. Tyramine showed profound cytotoxic and genotoxic effects, and induced cell cycle arrest and necrosis in HCT 116 cells. Our observation of tyramine-induced cytotoxicity is consistent with previous studies, where tyramine was tested at higher concentrations (e.g., 14.58 mM) using a different colonic cell line, namely, HT29 [79, 80]. In our study, tyramine started to induce significant cytotoxic effects at 0.8 mM. These differences in cytotoxic doses could be related to different cell lines used in these experiments. Moreover, we identified necrosis as the main form of cell death 24-h post-tyramine treatment, which is in agreement with a previous study on the HT29 cell line [80]. In contrast, apoptosis-involved genes, such as Caspase 7 (CASP7) and BCL2 binding component 3 (BBC3) were upregulated in a short tyramine treatment (6 h) at a high dose (14.58 mM) in HT29 cells [27]. These data may suggest apoptosis is an acute cell response to tyramine and it shifts to necrosis as time and concentrations increase, as supported by our data in which apoptosis alone was significantly reduced at higher treatment concentrations.

Genomic instability and high proliferation of cells are known risk factors for cancer [48]. Our data showed that tyramine increased the frequency of γH2AX + cells in tyramine-treated HCT 116 cells and promoted the frequency of MN formation. A previous study in HT29 cells reported altered expression levels of genes involved in DNA damage and repair pathways after tyramine treatment, such as ERCC3, GADD45G, POLB, and PPP1R15A [27]. Furthermore, we found that tyramine downregulated the expression of histones, whereas genes involved in oxidation/reduction reactions and oxidative stress response (e.g., AKR1B10, CYP4F3, CYP4F11, OSGIN1) were upregulated. Pathways involved in chromatin organization and assembly were reduced, suggesting genomic instability in response to tyramine treatment. This finding was further supported by our observation that the frequency of γH2AX + cells was significantly higher in the gut tissue of tyramine-treated WT mice compared to water-treated WT mice. This evidence indicates that tyramine can induce DNA damage in healthy mice, suggesting its role in increasing CRC risk *via* its genotoxic effect. Furthermore, we observed that tyramine treatment resulted in cell cycle arrest at G2 when DNA damage occurred. It is likely that cells undergo an arrest of the cell cycle allowing for DNA repair to occur [81]. We also found an increase in the percentage of Ki67 + cells and impaired mitochondria-related functions following tyramine treatment both in vitro and in vivo. These observations are consistent with previously reported data demonstrating that biological pathways associated with DNA damage repair and proliferation were enhanced at the early stages of CRC, whereas processes related to mitochondrial function were reduced [82]. With the phenotypical observation of the development of adenoma formation in WT mice receiving tyramine, these data, as a whole, prompt us to conclude that tyramine increases CRC risk by increasing DNA damage and cell proliferation.

Inflammation and impaired gut epithelial barrier function are also risk factors for CRC [83]. We found that tyramine treatment increased the gene expression levels of *IL-17A* in the colon and *Ngp* in the ileum in contrast to water-treated *Apc*^*Min/*+^ mice. The Ngp protein belongs to the cystatin family with a critical role in the regulation of inflammatory response [84]. Ly6G + cells identified as neutrophils were also in greater abundance in the ileum of tyramine-treated *Apc*^*Min/*+^ mice. These data suggest that tyramine promotes an inflammatory environment in the gut mucosa. Innate immune pathways driving inflammation are increased during CRC onset in the epithelium [82]. Loss of *Apc* induces disruption of the epithelial barrier activating tumor-associated myeloid cells by components of the gut microbiota, which regulate IL-17 production [85]. Furthermore, we observed higher expression levels of several genes involved in epithelial barrier function, such as defensins and C-type lectins (*Defb14* and *Clec2f*) in the intestinal tissues of tyramine-treated WT mice compared to water-treated WT mice. These data collectively suggest that tyramine contributes to epithelial barrier impairment and drives an inflammatory response.

Another observation was that tyramine increased colonic tumor size and exacerbated the appearance of *Apc*^*Min/*+^ mice compared to water-treated mice. RNAseq analysis revealed that tyramine impacted cell division and disrupted the extracellular matrix organization in *Apc*^*Min/*+^ and wild-type mice. Extracellular matrix composition and structure are deregulated in CRC to promote tumor growth [86]. Changes in the extracellular matrix promoted the proliferation of cancer cells [86]. Enrichment in extracellular matrix organization pathways has also been previously reported in a colitis-associated CRC model indicating their importance in tumor initiation [87]. Furthermore, we observed that in the tumor microenvironment NK cells were found to be lower and PMN-MDSCs displayed an increasing trend. Previous studies have shown that NK cells were rarely detected in colonic tumors from *Apc*^*Min/*+^ mice [88], whereas PMN-MDSCs accumulation inhibited anti-tumor T-cell responses [89]. These immune responses are consistent with a previously reported transcriptomic analysis of CRC patients, showing that anti-tumor immune responses including activation of NK cells and leukocyte mediated cytotoxicity were downregulated in the initial stages of normal to adenoma transition [90]. These observations suggest that tyramine has a potential to deregulate the extracellular matrix and suppress an effective anti-tumor immune response, favoring tumor progression.

In summary, the study findings support our hypothesis that tyramine derived from the diet and/or bacterial metabolism of tyrosine facilitates tumorigenesis and CRC development, particularly under circumstances where there is a genetic predisposition as is the case with a mutated *APC* gene. It is interesting to speculate whether a similar situation exists with other CRC genetic predispositions such as mutant RAS or p53, which warrant further investigation. Furthermore, it is important to note that the gut microbiome of patients with RYGB or IBD exhibits similar characteristics, including high fecal abundances of *Enterobacteriaceae* and *Enterococcaceae* and fecal concentrations of tyramine. Our investigation into the tyramine production of these bacterial isolates and the role of tyramine in increasing CRC risk could provide valuable insights for IBD research. These findings could also be valuable in the development of nutritional prevention strategies to reduce the risk of CRC.

## Supplementary Information

Supplementary Material 1.

## Data Availability

The RNAseq datasets analyzed during the current study were deposited in the GEO (Gene Expression Omnibus) repository of the National Center for Biotechnology Information (NCBI) under the SuperSeries GSE235266 including the GSE235264 and the GSE233876 study accession numbers. Metabolite data have been deposited to MetaboLights database under study number MTBLS8418.

## References

[1] Arterburn DE, Telem DA, Kushner RF, Courcoulas AP. benefits and risks of bariatric surgery in adults: a review. JAMA. 2020;324:879–87. 32870301 10.1001/jama.2020.12567

[2] Bruno DS, Berger NA. Impact of bariatric surgery on cancer risk reduction. Ann Transl Med. 2020;8:S13. 32309417 10.21037/atm.2019.09.26 PMC7154324

[3] Derogar M, et al. Increased risk of colorectal cancer after obesity surgery. Ann Surg. 2013;258:983–8. 23470581 10.1097/SLA.0b013e318288463a

[4] Tao W, et al. Colon and rectal cancer risk after bariatric surgery in a multicountry Nordic cohort study. Int J Cancer. 2020;147:728–35. 31797382 10.1002/ijc.32770

[5] Ostlund MP, Lu Y, Lagergren J. Risk of obesity-related cancer after obesity surgery in a population-based cohort study. Ann Surg. 2010;252:972–6. 20571362 10.1097/SLA.0b013e3181e33778

[6] Sainsbury A, et al. Increased colorectal epithelial cell proliferation and crypt fission associated with obesity and roux-en-Y gastric bypass. Cancer Epidemiol Biomarkers Prev. 2008;17:1401–10. 18559555 10.1158/1055-9965.EPI-07-2874

[7] Guo Y, et al. Modulation of the gut microbiome: a systematic review of the effect of bariatric surgery. Eur J Endocrinol. 2018;178:43–56. 28916564 10.1530/EJE-17-0403

[8] Li JV, et al. Roux-en-Y gastric bypass-induced bacterial perturbation contributes to altered host-bacterial co-metabolic phenotype. Microbiome. 2021;9:139. 34127058 10.1186/s40168-021-01086-x PMC8201742

[9] Li JV, et al. Metabolic surgery profoundly influences gut microbial-host metabolic cross-talk. Gut. 2011;60:1214–23. 21572120 10.1136/gut.2010.234708 PMC3677150

[10] A. P. Liou et al., Conserved shifts in the gut microbiota due to gastric bypass reduce host weight and adiposity. Sci Transl Med. 2013;5:178ra141. 10.1126/scitranslmed.3005687 PMC3652229 23536013

[11] Seyfried F, et al. Roux-en-Y gastric bypass surgery in Zucker rats induces bacterial and systemic metabolic changes independent of caloric restriction-induced weight loss. Gut Microbes. 2021;13:1–20. 10.1080/19490976.2021.1875108 PMC7872092 33535876

[12] Tremaroli V, et al. Roux-en-Y gastric bypass and vertical banded gastroplasty induce long-term changes on the human gut microbiome contributing to fat mass regulation. Cell Metab. 2015;22:228–38. 26244932 10.1016/j.cmet.2015.07.009 PMC4537510

[13] West KA, et al. Longitudinal metabolic and gut bacterial profiling of pregnant women with previous bariatric surgery. Gut. 2020;69:1452–9. 31964751 10.1136/gutjnl-2019-319620 PMC7398482

[14] Ilhan ZE, et al. Temporospatial shifts in the human gut microbiome and metabolome after gastric bypass surgery. NPJ Biofilms Microbiomes. 2020;6:12. 32170068 10.1038/s41522-020-0122-5 PMC7070067

[15] Louis P, Hold GL, Flint HJ. The gut microbiota, bacterial metabolites and colorectal cancer. Nat Rev Microbiol. 2014;12:661–72. 25198138 10.1038/nrmicro3344

[16] Kaur H, Das C, Mande SS. In silico analysis of putrefaction pathways in bacteria and its implication in colorectal cancer. Front Microbiol. 2017;8:2166. 29163445 10.3389/fmicb.2017.02166 PMC5682003

[17] Wirbel J, et al. Meta-analysis of fecal metagenomes reveals global microbial signatures that are specific for colorectal cancer. Nat Med. 2019;25:679–89. 30936547 10.1038/s41591-019-0406-6 PMC7984229

[18] A. Lo Presti et al., Fecal and mucosal microbiota profiling in irritable bowel syndrome and inflammatory bowel disease. Front Microbiol. 2019;10:1655. 10.3389/fmicb.2019.01655 PMC6650632 31379797

[19] Santoru ML, et al. Cross sectional evaluation of the gut-microbiome metabolome axis in an Italian cohort of IBD patients. Sci Rep. 2017;7:9523. 28842640 10.1038/s41598-017-10034-5 PMC5573342

[20] A. Vich Vila et al., Faecal metabolome and its determinants in inflammatory bowel disease. Gut. 2023;72:1472–1485. 10.1136/gutjnl-2022-328048 PMC10359577 36958817

[21] Salahshouri P, et al. A metabolic model of intestinal secretions: the link between human microbiota and colorectal cancer progression. Metabolites. 2021;11:456. 10.3390/metabo11070456 PMC8303431 34357350

[22] Alvarez MA, Moreno-Arribas MV. The problem of biogenic amines in fermented foods and the use of potential biogenic amine-degrading microorganisms as a solution. Trends Food Sci Technol. 2014;39:146–55.

[23] K. Bugda Gwilt et al., Actions of trace amines in the brain-gut-microbiome axis via trace amine-associated receptor-1 (TAAR1). Cellular and Molecular Neurobiology. 2019;40:191–201. 10.1007/s10571-019-00772-7 PMC11448870 31836967

[24] Bonnin-Jusserand M, Grandvalet C, Rieu A, Weidmann S, Alexandre H. Tyrosine-containing peptides are precursors of tyramine produced by Lactobacillus plantarum strain IR BL0076 isolated from wine. BMC Microbiol. 2012;12:199. 22963406 10.1186/1471-2180-12-199 PMC3492074

[25] Perez M, et al. Tyramine biosynthesis is transcriptionally induced at low pH and improves the fitness of Enterococcus faecalis in acidic environments. Appl Microbiol Biotechnol. 2015;99:3547–58. 25529314 10.1007/s00253-014-6301-7

[26] de Almeida CV, Taddei A, Amedei A. The controversial role of Enterococcus faecalis in colorectal cancer. Therap Adv Gastroenterol. 2018;11:1756284818783606. 10.1177/1756284818783606 PMC6044108 30013618

[27] Del Rio B, et al. An altered gene expression profile in tyramine-exposed intestinal cell cultures supports the genotoxicity of this biogenic amine at dietary concentrations. Sci Rep. 2018;8:17038. 30451877 10.1038/s41598-018-35125-9 PMC6242974

[28] S. H. Duncan, G. L. Hold, H. J. M. Harmsen, C. S. Stewart, H. J. Flint, Growth requirements and fermentation products of Fusobacterium prausnitzii, and a proposal to reclassify it as Faecalibacterium prausnitzii gen. nov., comb. nov. Int J Syst Evol Microbiol. 2010;52:2141–2146. 10.1099/00207713-52-6-2141 12508881

[29] Mahenthiralingam E, Campbell ME, Foster J, Lam JS, Speert DP. Random amplified polymorphic DNA typing of Pseudomonas aeruginosa isolates recovered from patients with cystic fibrosis. J Clin Microbiol. 1996;34:1129–35. 8727889 10.1128/jcm.34.5.1129-1135.1996 PMC228968

[30] Jameson E, et al. Anaerobic choline metabolism in microcompartments promotes growth and swarming of Proteus mirabilis. Environ Microbiol. 2016;18:2886–98. 26404097 10.1111/1462-2920.13059 PMC5026066

[31] Dona AC, et al. Precision high-throughput proton NMR spectroscopy of human urine, serum, and plasma for large-scale metabolic phenotyping. Anal Chem. 2014;86:9887–94. 25180432 10.1021/ac5025039

[32] Cloarec O, et al. Statistical total correlation spectroscopy: an exploratory approach for latent biomarker identification from metabolic 1H NMR data sets. Anal Chem. 2005;77:1282–9. 15732908 10.1021/ac048630x

[33] D. S. Wishart et al., HMDB 5.0: the human metabolome database for 2022. Nucleic Acids Res. 2022;50:D622-D631. 10.1093/nar/gkab1062 PMC8728138 34986597

[34] Patel SA, et al. Interleukin-6 mediated upregulation of CYP1B1 and CYP2E1 in colorectal cancer involves DNA methylation, miR27b and STAT3. Br J Cancer. 2014;111:2287–96. 25333344 10.1038/bjc.2014.540 PMC4264448

[35] Moser AR, Pitot HC, Dove WF. A dominant mutation that predisposes to multiple intestinal neoplasia in the mouse. Science. 1990;247:322–4. 2296722 10.1126/science.2296722

[36] Su LK, et al. Multiple intestinal neoplasia caused by a mutation in the murine homolog of the APC gene. Science. 1992;256:668–70. 1350108 10.1126/science.1350108

[37] Ju J, et al. Inhibition of intestinal tumorigenesis in Apcmin/+ mice by (-)-epigallocatechin-3-gallate, the major catechin in green tea. Cancer Res. 2005;65:10623–31. 16288056 10.1158/0008-5472.CAN-05-1949

[38] Curio S, et al. NKG2D signaling regulates IL-17A-producing γδT cells in mice to promote cancer progression. Discovery Immunology. 2022;1:kyac002. 10.1093/discim/kyac002 PMC9580227 36277678

[39] Dobin A, Gingeras TR. Mapping RNA-seq reads with STAR. Curr Protoc Bioinformatics. 2015;51:11.14.1-11.14.19. 10.1002/0471250953.bi1114s51 PMC4631051 26334920

[40] Liao Y, Smyth GK, Shi W. featureCounts: an efficient general purpose program for assigning sequence reads to genomic features. Bioinformatics. 2014;30:923–30. 24227677 10.1093/bioinformatics/btt656

[41] Love MI, Huber W, Anders S. Moderated estimation of fold change and dispersion for RNA-seq data with DESeq2. Genome Biol. 2014;15:550. 25516281 10.1186/s13059-014-0550-8 PMC4302049

[42] M. I. Love, S. Anders, V. Kim, W. Huber, RNA-Seq workflow: gene-level exploratory analysis and differential expression. F1000Res. 2015;4:1070. 10.12688/f1000research.7035.1 PMC4670015 26674615

[43] Young MD, Wakefield MJ, Smyth GK, Oshlack A. Gene ontology analysis for RNA-seq: accounting for selection bias. Genome Biol. 2010;11:R14. 20132535 10.1186/gb-2010-11-2-r14 PMC2872874

[44] Subramanian A, et al. Gene set enrichment analysis: a knowledge-based approach for interpreting genome-wide expression profiles. Proc Natl Acad Sci U S A. 2005;102:15545–50. 16199517 10.1073/pnas.0506580102 PMC1239896

[45] Luo W, Friedman MS, Shedden K, Hankenson KD, Woolf PJ. GAGE: generally applicable gene set enrichment for pathway analysis. BMC Bioinformatics. 2009;10:161. 19473525 10.1186/1471-2105-10-161 PMC2696452

[46] Ginestet C. ggplot2: Elegant Graphics for Data Analysis. J R Stat Soc a Stat. 2011;174:245–245.

[47] Liu Z, et al. A subset of Roux-en-Y gastric bypass bacterial consortium colonizes the gut of nonsurgical rats without inducing host-microbe metabolic changes. mSystems. 2020;5:e01047–20. 10.1128/mSystems.01047-20 PMC8579838 33293406

[48] Negrini S, Gorgoulis VG, Halazonetis TD. Genomic instability–an evolving hallmark of cancer. Nat Rev Mol Cell Biol. 2010;11:220–8. 20177397 10.1038/nrm2858

[49] Mah LJ, El-Osta A, Karagiannis TC. gammaH2AX: a sensitive molecular marker of DNA damage and repair. Leukemia. 2010;24:679–86. 20130602 10.1038/leu.2010.6

[50] Luzhna L, Kathiria P, Kovalchuk O. Micronuclei in genotoxicity assessment: from genetics to epigenetics and beyond. Front Genet. 2013;4:131. 23874352 10.3389/fgene.2013.00131 PMC3708156

[51] Baldwin EL, Osheroff N. Etoposide, topoisomerase II and cancer. Curr Med Chem Anticancer Agents. 2005;5:363–72. 16101488 10.2174/1568011054222364

[52] Sumei S, et al. Hypermethylation of DHRS3 as a novel tumor suppressor involved in tumor growth and prognosis in gastric cancer. Front Cell Dev Biol. 2021;9:624871. 33553182 10.3389/fcell.2021.624871 PMC7859350

[53] Zapater JL, Lednovich KR, Khan MW, Pusec CM, Layden BT. Hexokinase domain-containing protein-1 in metabolic diseases and beyond. Trends Endocrinol Metab. 2022;33:72–84. 34782236 10.1016/j.tem.2021.10.006 PMC8678314

[54] You S, et al. SLC7A1 overexpression is involved in energy metabolism reprogramming to induce tumor progression in epithelial ovarian cancer and is associated with immune-infiltrating cells. J Oncol. 2022;2022:5864826. 36131790 10.1155/2022/5864826 PMC9484923

[55] Schmitt M, Greten FR. The inflammatory pathogenesis of colorectal cancer. Nat Rev Immunol. 2021;21:653–67. 33911231 10.1038/s41577-021-00534-x

[56] Li J, et al. Ferroptosis: past, present and future. Cell Death Dis. 2020;11:88. 32015325 10.1038/s41419-020-2298-2 PMC6997353

[57] Linseisen J, et al. Dietary intake of different types and characteristics of processed meat which might be associated with cancer risk–results from the 24-hour diet recalls in the European Prospective Investigation into Cancer and Nutrition (EPIC). Public Health Nutr. 2006;9:449–64. 16870017 10.1079/phn2005861

[58] Andersen G, Marcinek P, Sulzinger N, Schieberle P, Krautwurst D. Food sources and biomolecular targets of tyramine. Nutr Rev. 2019;77:107–15. 30165672 10.1093/nutrit/nuy036

[59] Paulsen P, Grossgut R, Bauer F, Rauscher-Gabernig E. Estimates of maximum tolerable levels of tyramine content in foods in Austria. J Food Nutr Res. 2012;51:52–9.

[60] Babusyte A, Kotthoff M, Fiedler J, Krautwurst D. Biogenic amines activate blood leukocytes via trace amine-associated receptors TAAR1 and TAAR2. J Leukoc Biol. 2013;93:387–94. 23315425 10.1189/jlb.0912433

[61] K. Bugda Gwilt, N. Olliffe, R. A. Hoffing, G. M. Miller, Trace amine associated receptor 1 (TAAR1) expression and modulation of inflammatory cytokine production in mouse bone marrow-derived macrophages: a novel mechanism for inflammation in ulcerative colitis. Immunopharmacol Immunotoxicol. 2019;41:577–585. 10.1080/08923973.2019.1672178 31570011

[62] Hurtado CG, Wan F, Housseau F, Sears CL. Roles for interleukin 17 and adaptive immunity in pathogenesis of colorectal cancer. Gastroenterology. 2018;155:1706–15. 30218667 10.1053/j.gastro.2018.08.056 PMC6441974

[63] S. Curio et al., NKG2D signaling regulates IL-17A-producing gammadeltaT cells in mice to promote cancer progression. Discov Immunol. 2022;1:kyac002. 10.1093/discim/kyac002 PMC9580227 36277678

[64] Chae WJ, et al. Ablation of IL-17A abrogates progression of spontaneous intestinal tumorigenesis. Proc Natl Acad Sci U S A. 2010;107:5540–4. 20212110 10.1073/pnas.0912675107 PMC2851824

[65] Chiossone L, Dumas PY, Vienne M, Vivier E. Natural killer cells and other innate lymphoid cells in cancer. Nat Rev Immunol. 2018;18:671–88. 30209347 10.1038/s41577-018-0061-z

[66] Dhar P, Wu JD. NKG2D and its ligands in cancer. Curr Opin Immunol. 2018;51:55–61. 29525346 10.1016/j.coi.2018.02.004 PMC6145810

[67] Gabrilovich DI, Ostrand-Rosenberg S, Bronte V. Coordinated regulation of myeloid cells by tumours. Nat Rev Immunol. 2012;12:253–68. 22437938 10.1038/nri3175 PMC3587148

[68] Bronte V, et al. Recommendations for myeloid-derived suppressor cell nomenclature and characterization standards. Nat Commun. 2016;7:12150. 27381735 10.1038/ncomms12150 PMC4935811

[69] Jiang W, et al. Exhausted CD8+T cells in the tumor immune microenvironment: new pathways to therapy. Front Immunol. 2020;11:622509. 33633741 10.3389/fimmu.2020.622509 PMC7902023

[70] Elgundi Z, et al. Cancer metastasis: the role of the extracellular matrix and the heparan sulfate proteoglycan perlecan. Front Oncol. 2019;9:1482. 32010611 10.3389/fonc.2019.01482 PMC6978720

[71] Connil N, et al. Identification of the Enterococcus faecalis tyrosine decarboxylase operon involved in tyramine production. Appl Environ Microbiol. 2002;68:3537–44. 12089039 10.1128/AEM.68.7.3537-3544.2002 PMC126796

[72] F Özogul, Özogul Y. The ability of biogenic amines and ammonia production by single bacterial cultures. Eur Food Res Technol. 2007;225:385–394.

[73] Saito Y, Sato T, Nomoto K, Tsuji H. Identification of phenol- and p-cresol-producing intestinal bacteria by using media supplemented with tyrosine and its metabolites. FEMS Microbiol Ecol. 2018;94:fiy125. 10.1093/femsec/fiy125 PMC6424909 29982420

[74] Beloborodova NV, Khodakova AS, Bairamov IT, Olenin AY. Microbial origin of phenylcarboxylic acids in the human body. Biochemistry (Mosc). 2009;74:1350–5. 19961416 10.1134/s0006297909120086

[75] Smith EA, Macfarlane GT. Formation of phenolic and indolic compounds by anaerobic bacteria in the human large intestine. Microb Ecol. 1997;33:180–8. 9115181 10.1007/s002489900020

[76] H. M. H. Y. Hidehiko Kumagai, Formation of tyrosine phenol-lyase by bacteria. Agricultural and Biological Chemistry. 1970;34:3.

[77] Ponsky TA, Brody F, Pucci E. Alterations in gastrointestinal physiology after Roux-en-Y gastric bypass. J Am Coll Surg. 2005;201:125–31. 15978453 10.1016/j.jamcollsurg.2005.03.021

[78] Rafehi M, et al. Highly variable pharmacokinetics of tyramine in humans and polymorphisms in OCT1, CYP2D6, and MAO-A. Front Pharmacol. 2019;10:1297. 31736764 10.3389/fphar.2019.01297 PMC6831736

[79] Del Rio B, et al. The dietary biogenic amines tyramine and histamine show synergistic toxicity towards intestinal cells in culture. Food Chem. 2017;218:249–55. 27719906 10.1016/j.foodchem.2016.09.046

[80] Linares DM, et al. Comparative analysis of the in vitro cytotoxicity of the dietary biogenic amines tyramine and histamine. Food Chem. 2016;197:658–63. 26617000 10.1016/j.foodchem.2015.11.013

[81] Scully R, Panday A, Elango R, Willis NA. DNA double-strand break repair-pathway choice in somatic mammalian cells. Nat Rev Mol Cell Biol. 2019;20:698–714. 31263220 10.1038/s41580-019-0152-0 PMC7315405

[82] Roelands J, et al. Transcriptomic and immunophenotypic profiling reveals molecular and immunological hallmarks of colorectal cancer tumourigenesis. Gut. 2023;72:1326–39. 36442992 10.1136/gutjnl-2022-327608 PMC10314051

[83] Lichtenstern CR, Ngu RK, Shalapour S, Karin M. Immunotherapy, Inflammation and Colorectal Cancer. Cells. 2020;9:618. 10.3390/cells9030618 PMC7140520 32143413

[84] Liu K, et al. Neutrophilic granule protein (NGP) attenuates lipopolysaccharide-induced inflammatory responses and enhances phagocytosis of bacteria by macrophages. Cytokine. 2020;128:155001. 32035329 10.1016/j.cyto.2020.155001

[85] Grivennikov SI, et al. Adenoma-linked barrier defects and microbial products drive IL-23/IL-17-mediated tumour growth. Nature. 2012;491:254–8. 23034650 10.1038/nature11465 PMC3601659

[86] Li ZL, Wang ZJ, Wei GH, Yang Y, Wang XW. Changes in extracellular matrix in different stages of colorectal cancer and their effects on proliferation of cancer cells. World J Gastrointest Oncol. 2020;12:267–75. 32206177 10.4251/wjgo.v12.i3.267 PMC7081112

[87] Hammad A, et al. Transcriptome analysis of potential candidate genes and molecular pathways in colitis-associated colorectal cancer of Mkp-1-deficient mice. BMC Cancer. 2021;21:607. 34034704 10.1186/s12885-021-08200-0 PMC8152130

[88] Li Y, et al. Gut microbiota accelerate tumor growth via c-jun and STAT3 phosphorylation in APCMin/+ mice. Carcinogenesis. 2012;33:1231–8. 22461519 10.1093/carcin/bgs137

[89] Chun E, et al. CCL2 promotes colorectal carcinogenesis by enhancing polymorphonuclear myeloid-derived suppressor cell population and function. Cell Rep. 2015;12:244–57. 26146082 10.1016/j.celrep.2015.06.024 PMC4620029

[90] Hong Q, et al. Transcriptomic analyses of the adenoma-carcinoma sequence identify hallmarks associated with the onset of colorectal cancer. Front Oncol. 2021;11:704531. 34458146 10.3389/fonc.2021.704531 PMC8387103

