# Supplementary material for: Roux-en-Y gastric bypass-associated fecal tyramine promotes colon cancer risk via increased DNA damage, cell proliferation, and inflammation
Source: Microbiome. 2025 Feb 28;13:60. doi: 10.1186/s40168-025-02049-2 (PMC11869571; doi:10.1186/s40168-025-02049-2)
Supplement: Supplementary file 2 — Supplementary Material 1. [file 40168_2025_2049_MOESM1_ESM.docx]

**Supplementary information**

**Roux-en-Y gastric bypass-associated fecal tyramine promotes colon cancer risk via increased DNA damage, cell proliferation and inflammation**

Maria Glymenaki^1^, Sophie Curio^2,†^, Smeeta Shrestha^3^, Qi Zhong^1^, Laura Rushton^1, #^, Rachael Barry^1^, Mona El-Bahrawy^1^, Julian R. Marchesi^1^, Yulan Wang^3^, Nigel J. Gooderham^1^, Nadia Guerra^2^, Jia V. Li^1,^*

**Affiliations:**

^1^Division of Digestive Diseases, Department of Metabolism, Digestion and Reproduction, Imperial College London, UK

^2^Department of Life Sciences, Imperial College London, UK

^3^Singapore Phenome Center, Lee Kong Chian School of Medicine, Nanyang Technological University, Singapore 308232, Singapore

† Present Address: The University of Queensland Frazer Institute, The University of Queensland, Woolloongabba, QLD 4102, Australia

^#^ Present Address: Department for Environment Food and Rural Affairs, UK

***Corresponding Author**: Jia V. Li, Section of Nutrition, Division of Digestive Diseases, Department of Metabolism, Digestion and Reproduction, Faculty of Medicine, Imperial College London, W12 0NN, London, United Kingdom. Phone: +44 (0)20 7594 3230; E-mail: [jia.li@imperial.ac.uk](mailto:jia.li@imperial.ac.uk).

**Methods**

**Tissue dissociation and flow cytometry**

For the analysis of tumor-infiltrating leukocytes in the gut, tumors were macroscopically delineated, collected, cut into small pieces and digested with Collagenase IV (150 U/ml, Sigma) and DNase I (50 U/ml, Sigma) for 30 minutes at 37°C in the presence of RPMI medium (Gibco) supplemented with 5% FBS (Gibco). They were subsequently washed in PBS containing 0.5% FBS and 50 mM EDTA, filtered on a 100-μm cell strainer and centrifuged (800 g, 10 min, 4°C). Cell pellets were washed in PBS, and incubated with Rat IgG2a kappa Isotype Control (eBioscience) to block Fc receptors and Zombie UV Fixable Viability dye (Biolegend) for 20 minutes at 4°C. Cells were washed with 0.5% PBS-BSA, stained with a cocktail of fluorophore- and biotin-conjugated antibodies (Supplementary Table 3) for 30 minutes at 4°C followed by a further staining step with Streptavidin-PerCP to detect biotinylated antibodies. Intracellular staining was performed using the Foxp3 / Transcription Factor Staining Buffer Set (eBioscience) according to manufacturer’s instructions. The relevant fluorescence-minus-one labelled cells, single stained appropriate isotype mAb, and unstained cell suspensions were used as controls. Anti-Rat and Anti-Hamster Ig, κ/Negative Control Compensation Particles Set (BD), Anti-Mouse Ig, κ/Negative Control Compensation Particles Set (BD), and MACS Comp Bead Kit, anti-REA (Miltenyi Biotec) were used for compensation setup. All samples were acquired on an Aurora spectral flow cytometer (Cytek) and analysed with Spectroflo (Cytek) and FlowJo version 10.7.1 or above (BD).

**Histology**

Ileum gut rolls taken at autopsy from *Apc^Min/+^* and WT mice were fixed overnight in formalin fixative solution containing 8% (v/v) formaldehyde, 0.9% (w/v) sodium chloride, 2% (v/v) glacial acetic acid and 0.5% (w/v) cetrimide in distilled water (dH_2_O). The fixed gut rolls were paraffin-embedded and sectioned at 5-μm thickness. The tissue slices were stained with haematoxylin and eosin (H&E) for histological assessment of tissue integrity. Dysplasia was scored as previously described[1]. Dysplasia scoring was performed blind by an expert pathologist. Slide pictures were taken using a NanoZoomer S210 slide scanner (Hamamatsu) and visualised using NDP.view2 software (Hamamatsu).

**Proton** **nuclear magnetic resonance (^1^H NMR) spectroscopic analysis of biological samples**

Spectra from cell media of HCT116 treated cells were obtained using a Bruker DRX 600 MHz spectrometer (Bruker, Rheinstetten, Germany) according to previously published standard operating procedures[2, 3]. Faecal samples from mice put in metabolic cages overnight to observe kinetics of tyramine absorption were weighted (~30-50 mg) and resuspended in D_2_O at a ratio 1:4. After three cycles of vortexing and sonication, 20 µL phosphate buffer (1.5 M potassium phosphate KH_2_PO_4_, which contained 100% D_2_O for magnetic field lock, 1% sodium 3-(trimethylsilyl)(2,2,3,3-d_4_)propionate (TSP) for spectral calibration, and 2 mM NaN_3_ (pH= 7.4) to avoid bacterial contamination) was added and samples were acquired for ^1^H NMR spectroscopy. Urine was mixed with the aforementioned phosphate buffer at a ratio of 9:1 (v:v). For the prepared faecal water and urine samples, a total of 128 scans were recorded into 64 k data points with a spectral width of 20 ppm at 300 K. Cell media were also mixed with the phosphate buffer and a total of 32 scans were recorded into 64 k data points with a spectral width of 20 ppm at 300 K.

**^1^H NMR data processing and multivariate statistical analysis**

^1^H NMR spectra were pre-processed in Topspin 3.3 (Bruker, Germany), including phasing, baseline correction and referencing to TSP peak at δ 0.0.  The processed data was imported into MATLAB (version 2017b) and the water peak region together with the regions containing TSP and noises were cut out. Remaining spectra were aligned using the recursive segment-wise peak alignment algorithm[4] to correct for chemical shift variations and normalized using the probabilistic quotient method[5]. Multivariate statistical analysis methods including principal components analysis (PCA)[6] and orthogonal signal correction−projection to latent structures−discriminant analysis (O-PLS-DA)[7] with a unit variance (UV) scaling method were carried out using SIMCA version 15 (Umetrics, Sartorius Stedim Biotech) and MATLAB (version 2017b, Mathworks). The validity and robustness of the O-PLS-DA models were assessed using 7-fold cross-validation and permutation testing. Statistical Total Correlation Spectroscopy (STOCSY) [8] was performed to assist in metabolite assignment along with the use of Chenomx NMR Suite (Chenomx, Canada), in-house and public datasets. ^1^H NMR spectra were submitted to MetaboLights [9].

**Table S1.** Chemical shift ranges of the selected peaks for the observed metabolites in ^1^H NMR spectra of bacterial culture medium.

| **Type** | **Metabolites** | **Chemical shift ranges (δ^1^H)** |
| --- | --- | --- |
| nutrient | glucose | 5.231-5.261 |
| nutrient | fumarate | 6.452-6.593 |
| nutrient | tyrosine | 7.2-7.209 |
| product | isopropanol | 1.169-1.174 |
| product | ethanol | 1.195-1.208 |
| product | lactate | 1.305-1.358 |
| product | putrescine | 1.759-1.8 |
| product | formate | 8.385-8.515 |
| product | acetate | 1.888-1.961 |
| product | succinate | 2.401-2.42 |
| product | malate | 4.274-4.329 |
| product | 2-oxoglutarate | 2.995-3.028 |
| product | tyramine | 7.213-7.24 |
| product | phenol | 6.98-7.015 |
| product | 4-hydroxyphenylacetate | 7.162-7.17 |
| product | 4-hydroxyphenyllactate | 6.859-6.864 |
| product | 4-hydroxyphenylpyruvate | 7.136-7.142 |

**Table S2.** Appearance scoring criteria.

| **Score** | **Behavior** |
| --- | --- |
| 0 | Alert, active, interested in surroundings |
| 1 | Alert, mildly less active |
| 2 | Alert, lethargic |
| 3 | Subdued, lethargic |
| 4 | Unresponsive even when provoked |
| **Score** | **Appearance** |
| 0 | Glossy coat, bright eyes |
| 2 | Piloerection/Starry coat |
| 2 | Mild hunched posture |
| 2 | Starry coat |
| **Score** | **Potential tumor adverse effects** |
| 2 | White paws (back or front) |
| 4 | White paws in both back and front legs |
| 4 | Blood in feces |
| 4 | Rectal prolapse |

**Table S3. A** list of antibodies used for flow cytometry of tumor infiltrating leukocytes.

| **Antigen** | **Clone** | **Concentration (μg/ml)** | **Fluorophore** | **Supplier** |
| --- | --- | --- | --- | --- |
| CD8 | 53-6.7 | 0.5 | BUV395 | BD |
| CD4 | GK1.5 | 0.5 | BUV496 | BD |
| Ly6G | 1A8 | 0.5 | BUV661 | BD |
| CD3 | 145-2C11 | 1 | BUV737 | BD |
| F4/80 | BM8 | 0.5 | Brilliant Violet 421 | Biolegend |
| IFN-γ | XMG1.2 | 2 | eFluor 450 | eBioscience |
| CD11b | M1/70.15.11.5 | n/a (dilution 1:800) | VioGreen | Miltenyi Biotec |
| Ly6C | HK1.4 | 0.025 | Brilliant Violet 605 | Biolegend |
| NK1.1 | PK136 | 1 | Brilliant Violet 650 | Biolegend |
| T-bet | 4B10 | 4 | Brilliant Violet 711 | Biolegend |
| B220 | RA3-6B2 | 0.25 | Brilliant Violet 785 | Biolegend |
| CD200R | REA850 | n/a (dilution 1:100) | Vio Bright B515 | Miltenyi Biotec |
| CD127 | A7R34 | 5 | FITC | eBioscience |
| CD107a (LAMP-1) | 1D4B | 5 | Biotin | Biolegend |
| CD206 (MMR) | C068C2 | 2 | PerCP/Cyanine5.5 | Biolegend |
| CD279 (PD-1) | J43 | 1 | PerCP-eFluor 710 | eBioscience |
| CD314 (NKG2D) | CX5 | *2* | PE | eBioscience |
| RORγt | B2D | 2 | PE-eFluor 610 | eBioscience |
| γδTCR | eBioGL3 (GL-3, GL3) | 0.5 | PE-Cyanine5 | eBioscience |
| IL-17 | TC11-18H10.1 | 2 | PE/Cyanine7 | Biolegend |
| Foxp3 | FJK-16s | 1 | APC | eBioscience |
| GzB | GB11 | 2 | Alexa Fluor 647 | BD |
| CD45 | 30-F11 | 0.5 | Alexa Fluor 700 | Biolegend |
| TNF-α | MP6-XT22 | 2 | APC/Cyanine7 | Biolegend |

**Results**

**Phenotypic characterization of tyramine-treated *Apc^Min/+^* and WT mice**

We did not observe a difference in body weight, food consumption and water intake throughout the study among tyramine-treated *Apc^Min/+^*, untreated *Apc^Min/+^*, tyramine-treated WT and untreated WT mice (Supplementary Fig. 13a-c). Spleen weight was significantly higher, and hematocrit was significantly lower in *Apc^Min/+^* mice compared to WT controls irrespective of the treatment regime (Supplementary Fig. 13d-e). These findings mirror previous results[10, 11] reported in *Apc^Min/+^* mice that develop anemia concomitant with tumor growth[12]. In contrast, the liver weight was comparable among the groups (Supplementary Fig. 13f). While the total gut length was comparable between tyramine-treated and untreated groups, a significantly shorter gut length was noted in tyramine-treated *Apc^Min/+^* mice compared to tyramine-treated WT mice (Supplementary Fig. 13g), suggesting a crosstalk between tyramine and genetic strains of mice, which result in this phenotypical change in the gut length. Colon length did not differ among the groups (Supplementary Fig. 13h). Histological analysis indicated a similar grade of dysplasia in the CRC-prone mice independent of treatment, and tyramine administration did not result in histological changes (Supplementary Fig. 13i).

Tyramine binds to a family of G-protein-coupled receptors, termed trace amine-associated receptors (TAARs), which are expressed in the intestine[13]. There is currently no information on TAAR1-mediated uptake of tyramine in gut epithelial cells, but it has been shown that tyramine can be absorbed through diffusion[14]. We did not detect tyramine in urine or feces when administered orally (Supplementary Fig. 14), suggesting that it is absorbed.

**Figures**

**
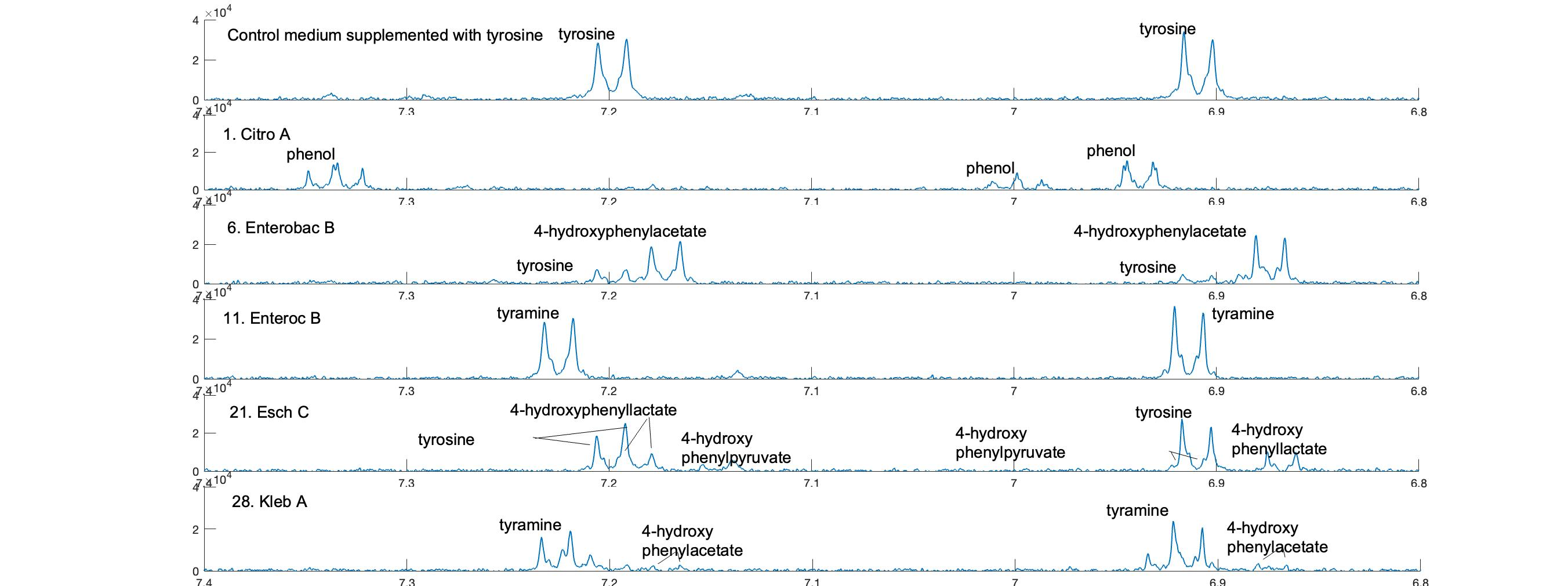
**

**Fig. S1** Partial representative ^1^H NMR spectra of bacterial culture medium samples. The media were derived from a control sample with no presence of bacteria, and bacterial culture with the following species number and types: 1. Citro A, 6. Enterobac B, 11. Enteroc B, 21. Esch C and 28. Kleb A. The numbers are correspondent to Table 1.


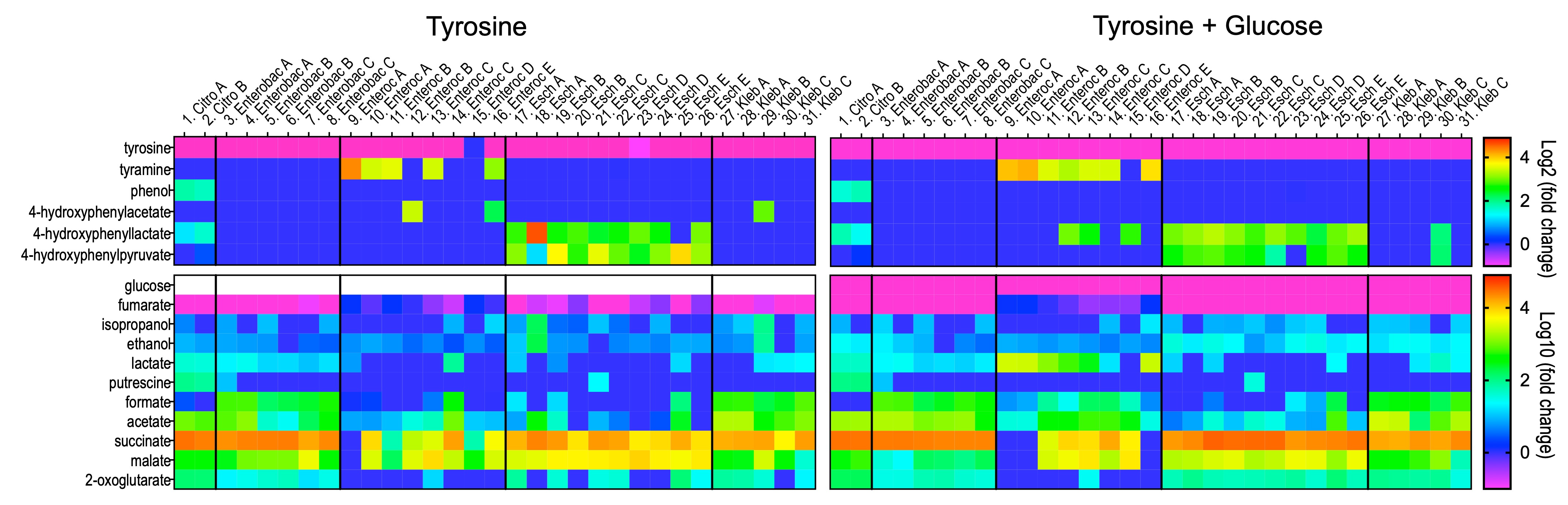


**Fig. S2** Metabolite production and utilization of the bacterial isolates cultured aerobically using simple base medium supplemented with tyrosine (left panel) or tyrosine and glucose (right panel). Fold changes of relative concentrations of metabolites in bacterial media to the control medium without bacteria were calculated. Negative values of log2 (fold change) or log10 (fold change) represent substrate utilization (e.g. glucose, fumarate and tyrosine), whereas positive values represent metabolite production (e.g. tyramine, succinate). Bacterial information can be found in Table 1.


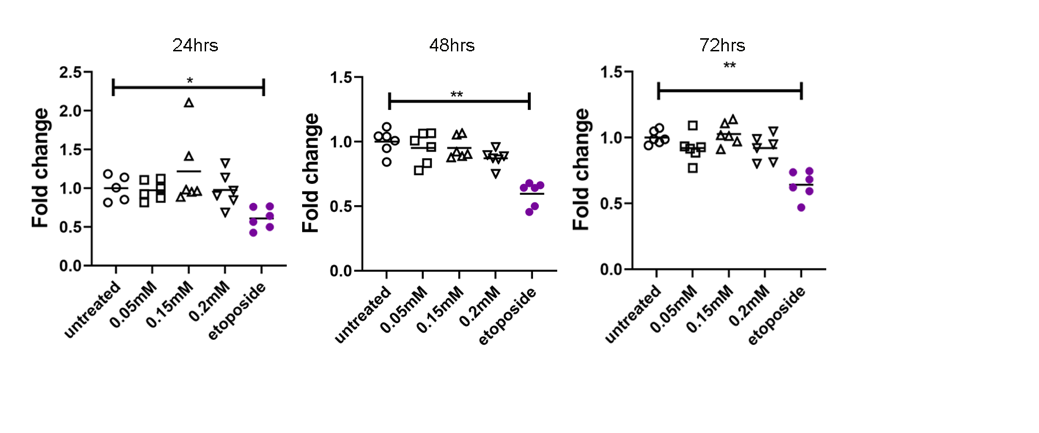


**Fig. S3** Tyramine treatment at low concentrations does not affect the viability of human colorectal cancer HCT116 cells. Fold change of cell viability measured as fluorescence intensity at 24, 48 and 72hrs in tyramine-treated groups relative to untreated after subtraction of background fluorescence in media. Data were pooled from two independent experiments (n=5-6 per group). Kruskal-Wallis test with Dunn’s multiple comparisons test. Shapes used for grouping throughout the graphs: circle, untreated; square, 0.05mM tyramine; triangle, 0.15 mM tyramine; inverted triangle, 0.2 mM tyramine; purple circle, etoposide. Data shown as means. *, p<0.05; **, p<0.01.


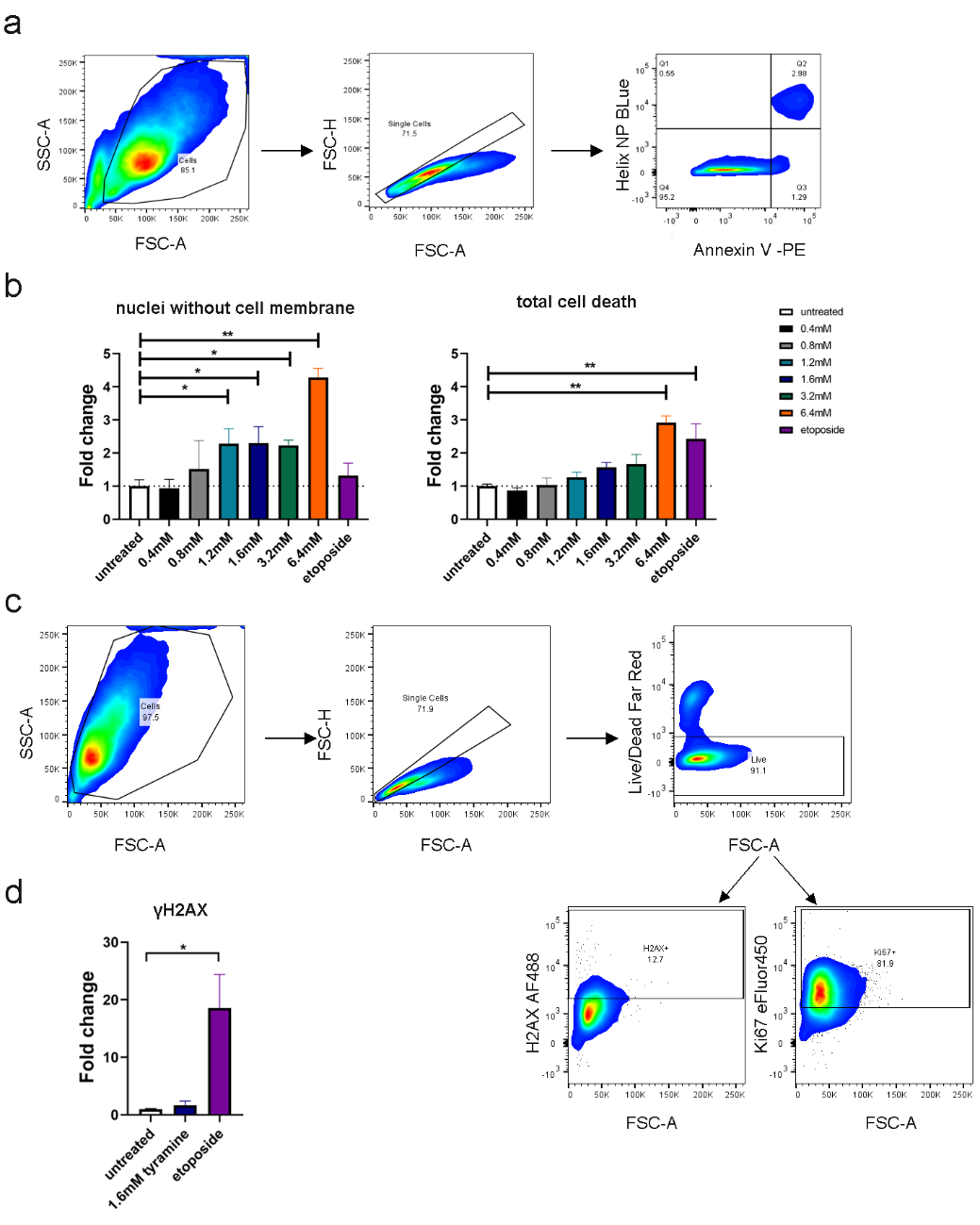


**Fig. S4** Tyramine causes cell death and incurs the appearance of DNA lesions. **a)** Gating strategy for Annexin V and Helix NP Blue DNA dye after 24 hrs of treatment with tyramine to distinguish apoptosis and necrosis using flow cytometry. **b)** Fold changes of nuclei without cell membrane and total dead cells using Annexin V and Helix NP Blue DNA dye after 24 hrs of treatment with tyramine relative to untreated control groups. Data from two independent experiments were pooled (n=6 per group). Kruskal-Wallis test followed by Dunn's multiple comparisons test. Color coding used: white, untreated; black, 0.4 mM tyramine; grey, 0.8 mM tyramine; petrol, 1.2 mM tyramine; dark blue, 1.6 mM tyramine; green, 3.2 mM tyramine; orange, 6.4mM tyramine; purple, etoposide. Data shown as means ± SD. *, p<0.05; **, p<0.01. **c)** Gating strategy for Ki67-positive and H2AX-positive cells as markers of proliferation and DNA damage respectively after 24 hrs of treatment with tyramine and acquisition using flow cytometry. **d)** Fold change of phosphorylated H2AX (γH2AX) positive cells treated with tyramine to the untreated group for 6 hrs. Etoposide was used as a positive control. Kruskal-Wallis test with Dunn’s multiple comparisons test (n=3 per group). Color coding used: white, untreated; dark blue, 1.6mM tyramine; purple, etoposide. Data shown as means ± SD. *, p<0.05.


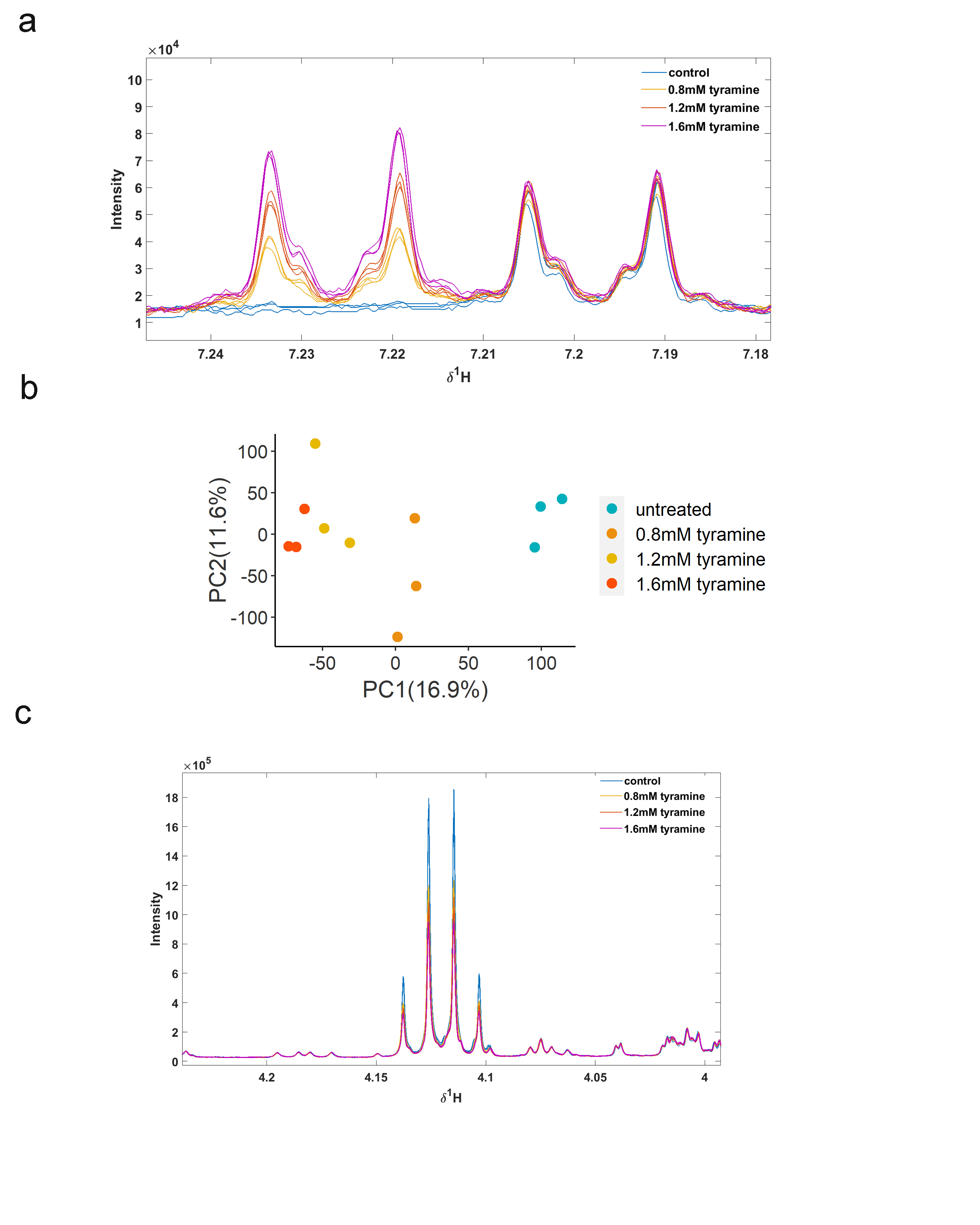


**Fig. S5** Proton nuclear magnetic resonance (^1^H NMR) analysis of media from untreated and tyramine-treated cells. **a)** Magnified spectrum region showing a duplet peak of tyramine at 7.23 ppm in 24 hr tyramine-treated samples and its absence in the untreated controls (n=6 for control and n=3 for 0.8 mM tyramine, 1.2 mM tyramine, and 1.6 mM tyramine). **b)** Principal Components Analysis (PCA) scores plot of ^1^H NMR spectra where the tyramine peaks have been removed to reveal differences in the metabolite profiles not confounded by the addition of tyramine. Media from tyramine-treated cells clustered separately than untreated controls. UV scaling was applied. **c)** Magnified spectrum region showing a quadruplet peak of lactate at 4.11 ppm. The peak is higher in controls compared to tyramine-treated samples (n=6 for control and n=3 for 0.8 mM tyramine, 1.2 mM tyramine, and 1.6 mM tyramine). Color coding used throughout the graphs: blue, untreated/control; orange, 0.8 mM tyramine; yellow, 1.2 mM tyramine; red, 1.6mM tyramine.


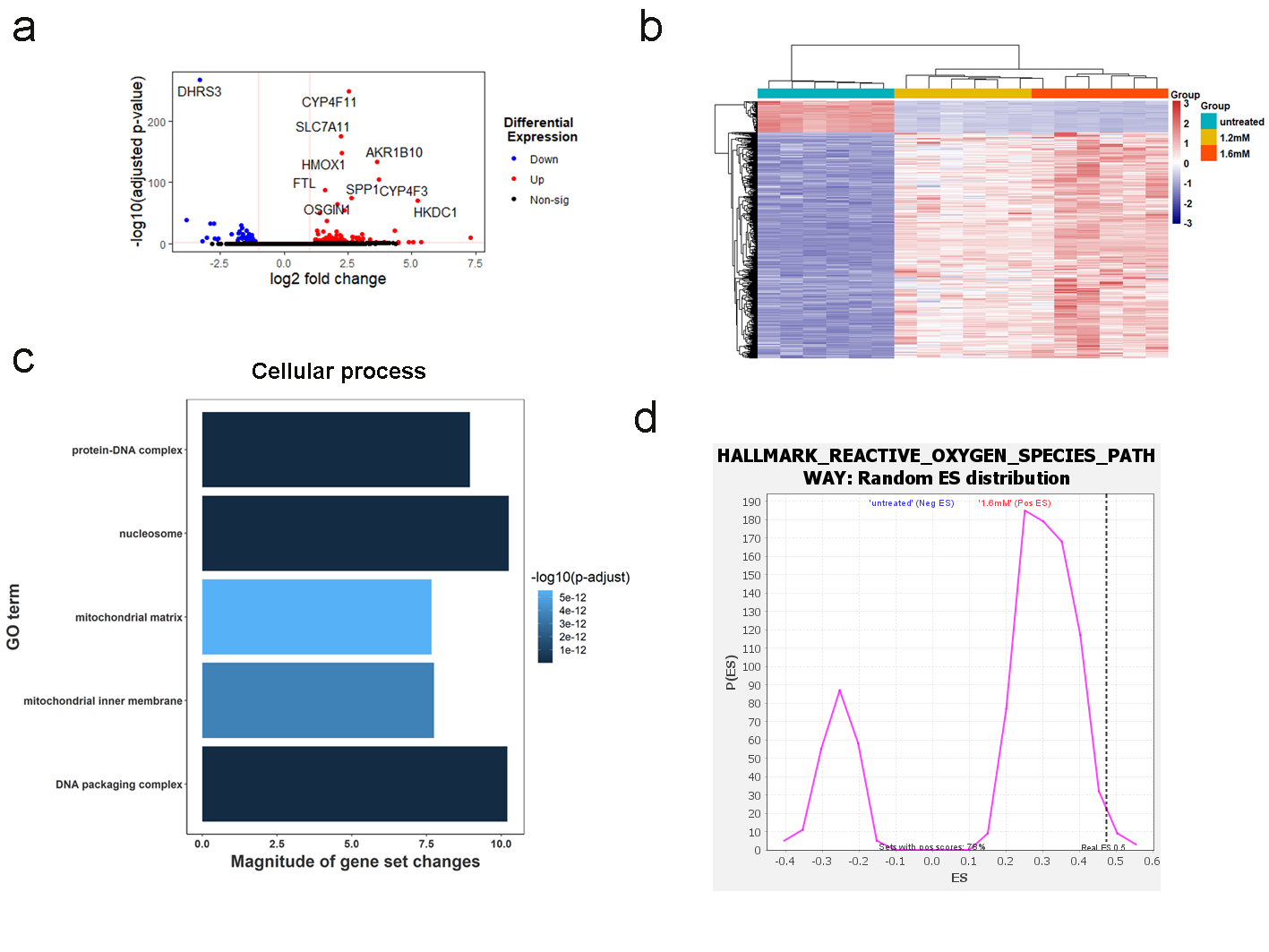


**Fig. S6** Tyramine regulates pathways involved in oxidative stress and mitochondrial function. **a)** Volcano plot of genes enriched or reduced in 1.2 mM tyramine treatment versus control. The dotted vertical lines enclose the minimum fold change for the most significant genes. A cut-off of >2 absolute value was used for fold change and <0.05 for FDR-corrected p-value to assign a gene as differentially expressed. The 10 most differentially expressed genes are shown on the plot. Abbreviations: AKR1B10, aldo-keto reductase family 1 member B10; CYP4F3, cytochrome P450 family 4 subfamily F member 3; CYP4F11, cytochrome P450 family 4 subfamily F member 11; DHRS3, dehydrogenase/reductase 3; FTL, ferritin light chain; GCLM, glutamate-cysteine ligase modifier subunit; HMOX1, heme oxygenase 1; OSGIN1, oxidative stress induced growth inhibitor 1; SLC7A11, solute carrier family 7 member 11; SPP1, secreted phosphoprotein 1. **b)** Heatmap of RNAseq data using z-scores of genes that are differentially expressed between 1.6 mM tyramine treatment group versus control. 239 differentially expressed genes are common in the pairwise comparisons of 1.6 mM tyramine versus control, and 1.2 mM tyramine versus control. Each column in the heatmap is an individual sample. **c)** Plot of the top 5 GO terms to visualize the cellular processes decreased in 1.6 mM tyramine treated cells compared to controls. FDR correction < 0.05 has been applied to the depicted GO terms. **d)** Random enrichment score (ES) distribution of the GSEA plot for Hallmark Reactive Oxygen Species Pathway.


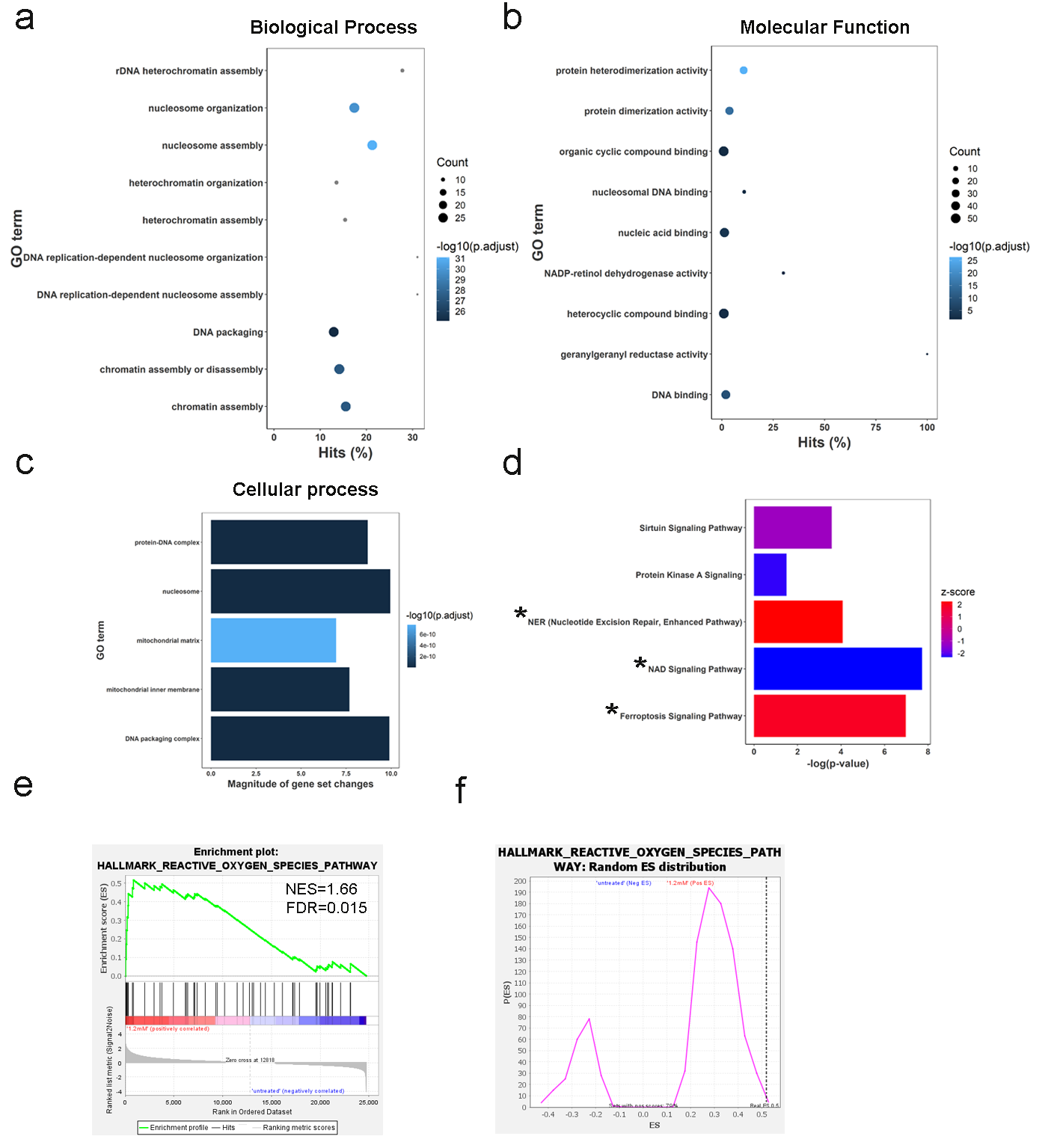


**Fig. S7** Tyramine incurs transcriptomic changes relevant to DNA and oxidative damage. Plots of the top 10 over-represented GO terms to visualize **a)** the biological processes, and **b)** molecular functions altered in 1.2 mM tyramine treated cells compared to controls. Hits reflect the proportion of differentially expressed genes in a given pathway. The number of altered genes is indicated by the size of the circle area, and the circle color represents the range of the corrected P value. FDR correction < 0.05 has been applied to the depicted GO terms. **c)** Plot of the top 5 GO terms to visualize the cellular processes decreased in 1.2mM tyramine treated cells compared to controls. FDR correction < 0.05 has been applied to the depicted GO terms. **d)** IPA analysis showing the top canonical pathways using z-scores in 1.2mM tyramine. Pathways with absolute z-score >1 are depicted. * denote the same pathways altered in 1.6mM tyramine. **e)** Gene set enrichment plot demonstrating correlation of gene sets involved in oxygen species pathway with 1.2 mM tyramine. Gene permutation was used. FDR correction <0.05 has been applied. NES, normalized enrichment score. **f)** Random enrichment score (ES) distribution of the GSEA plot for Hallmark Reactive Oxygen Species Pathway.

**
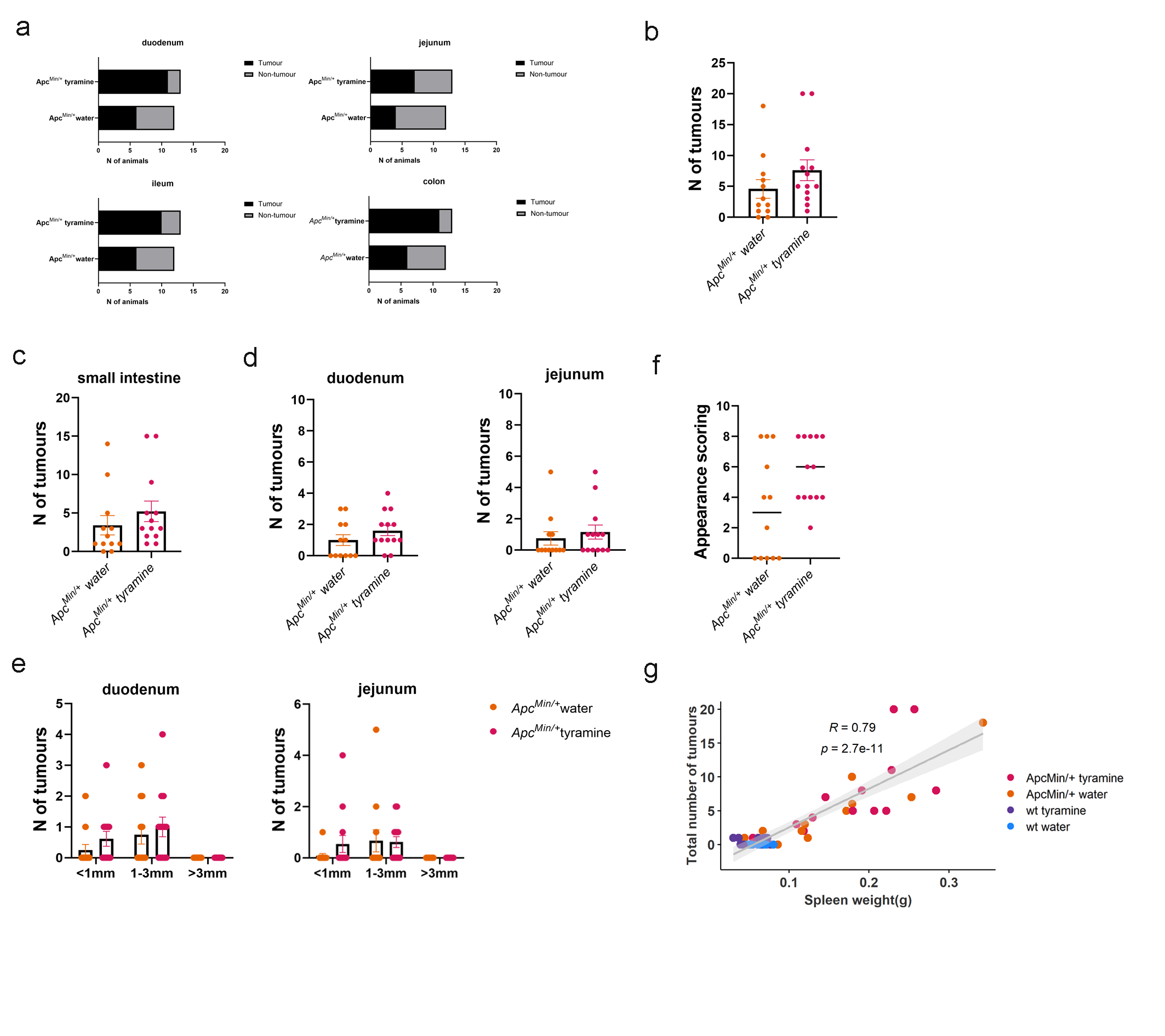
**

**Fig. S8** A higher number of *Apc^Min/+^* mice forms intestinal tumors in response to tyramine treatment compared to control *Apc^Min/+^* mice. **a)** Prevalence of tumors in different regions of the small intestine (duodenum, jejunum, ileum) and colon in tyramine-treated *Apc^Min/+^* and untreated *Apc^Min/+^* mice. **b)** Total tumor numbers of the small and large intestine were counted in 13-week-old tyramine-treated *Apc^Min/+^* mice (n=13) and untreated *Apc^Min/+^* mice (n=12 mice). **c)** Small intestine tumors, and **d)** tumor numbers from duodenum and jejunum. **e)** Tumor numbers based on tumor size in duodenum and jejunum. Tumors smaller than 1 mm, between 1–3 mm, and greater than 3 mm were counted separately. **f)** Appearance scoring in *Apc^Min/+^* mice at 13 weeks of age. Independent scoring by two people. **g)** Correlation of total number of tumors with spleen eight colored by the treatment group. Spearman correlation was applied. The confidence interval is shown as grey area at 95%. Orange, water-treated *Apc^Min/+^* mice; pink, tyramine-treated *Apc^Min/+^* mice; light blue, water-treated WT mice; purple, tyramine-treated WT mice. Data shown as means ± SEM except from f) where median is shown. Fisher's exact test in a). Mann-Whitney test was applied in b)- d), and f). Multiple Mann-Whitney tests followed by FDR approach for multiple comparisons in e). *, p<0.05.


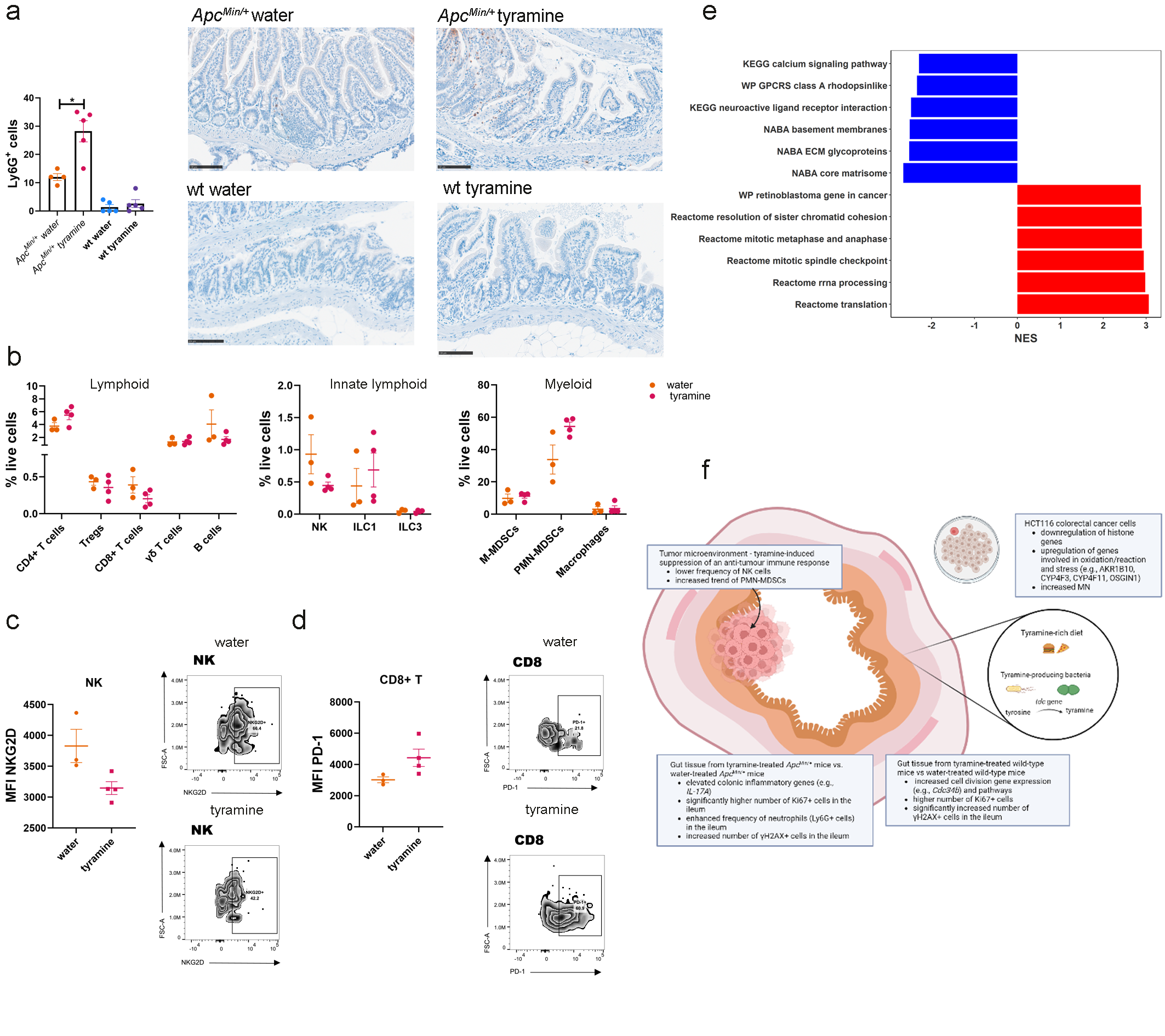


**Fig. S9** Tyramine dampens an anti-tumor immune response in the gut tissue and tumors. **a)** Number of Ly6G+ cells per slide in a gut roll from the ileum and representative immunohistochemistry images**.** Scale bar 100µm**.** **b)** Colonic tumor-infiltrating immune cells (lymphoid, innate lymphoid and myeloid) plotted as percentage of live non-autofluorescent cells in tyramine- and water-treated *Apc^Min/+^* mice at 13 weeks of age. **c)** Mean Fluorescence Intensity (MFI) of NKG2D in NK cells. Representative flow cytometry zebra plots for tyramine- and water- treated mice are also shown. **d)** MFI of PD-1 in CD8+ T cells and representative zebra plots. **e)** Tyramine treatment enhances mitosis and protein translation pathways in tumors from *Apc^Min/+^* mice. GSEA of the top-6 most significant downregulated and upregulated pathways in tumors from ileum. FDR correction <0.05 has been applied. NES, normalized enrichment score. **f)** Schematic on the effect of tyramine in CRC development. Orange, water-treated *Apc^Min/+^* mice; pink, tyramine-treated *Apc^Min/+^* mice; light blue, water-treated WT mice; purple, tyramine-treated WT mice. Mann-Whitney test for pairwise comparisons between tyramine-treated mice and water-treated *Apc^Min/+^* mice, and tyramine-treated and water-treated WT mice in a). Wilcoxon Rank Sum test was applied for between group comparisons followed by FDR method for adjusting p-values for multiple test correction in b)-d). Data shown as means ± SEM. Data represent two independent experiments (n=3-4 per group) in b)-d). *, p<0.05.

**Fig. S10** Flow cytometry gating strategy to delineate lymphoid, myeloid and innate lymphoid cells (ILCs). **a)** Gating of lymphoid cells: CD4+ T cells are identified as CD3+CD4+ after gating out dead cells and autofluorescent cells, CD8+ T cells are identified from the live non-autofluorescent gated cells as CD3+CD8+, and Tregs are identified from the viable non-autofluorescent cell gates as Foxp3+CD4+CD3+. Natural Killer cells (NK) are identified as CD3-NK1.1+. **b)** Gating of ILCs: Group 1 ILCs (ILC1s) and Group 3 ILCs (ILC3s) are identified after subsequent subgating out of the CD3-B220- and the Ly6C-Ly6G- as CD127+ T-bet+ and CD127+ RORγt+ cells respectively. B cells are identified from the viable non-autofluorescent gates as CD3-B220+. Gating of myeloid cells: Monocytic-myeloid derived suppressor cells (M-MDSCs) are identified form the CD11b+ gate as Ly6C+ and polymorphonuclear-MDSCs (PMN-MDSCs) as Ly6G+. Macrophages are identified as F4/80+ and depending on the expression of CD206, they are segregated to M1 (CD206-) and M2 (CD206+).


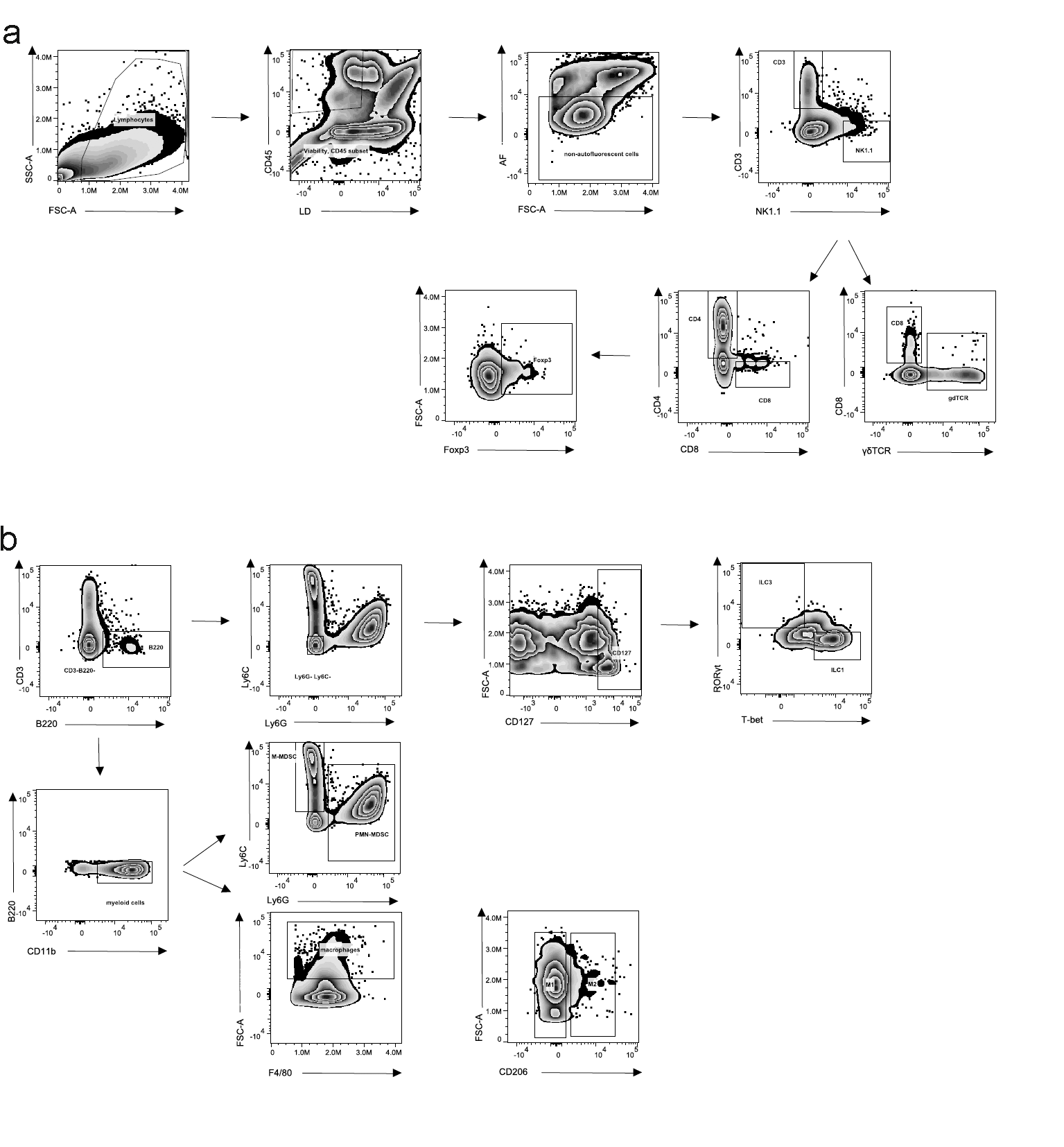

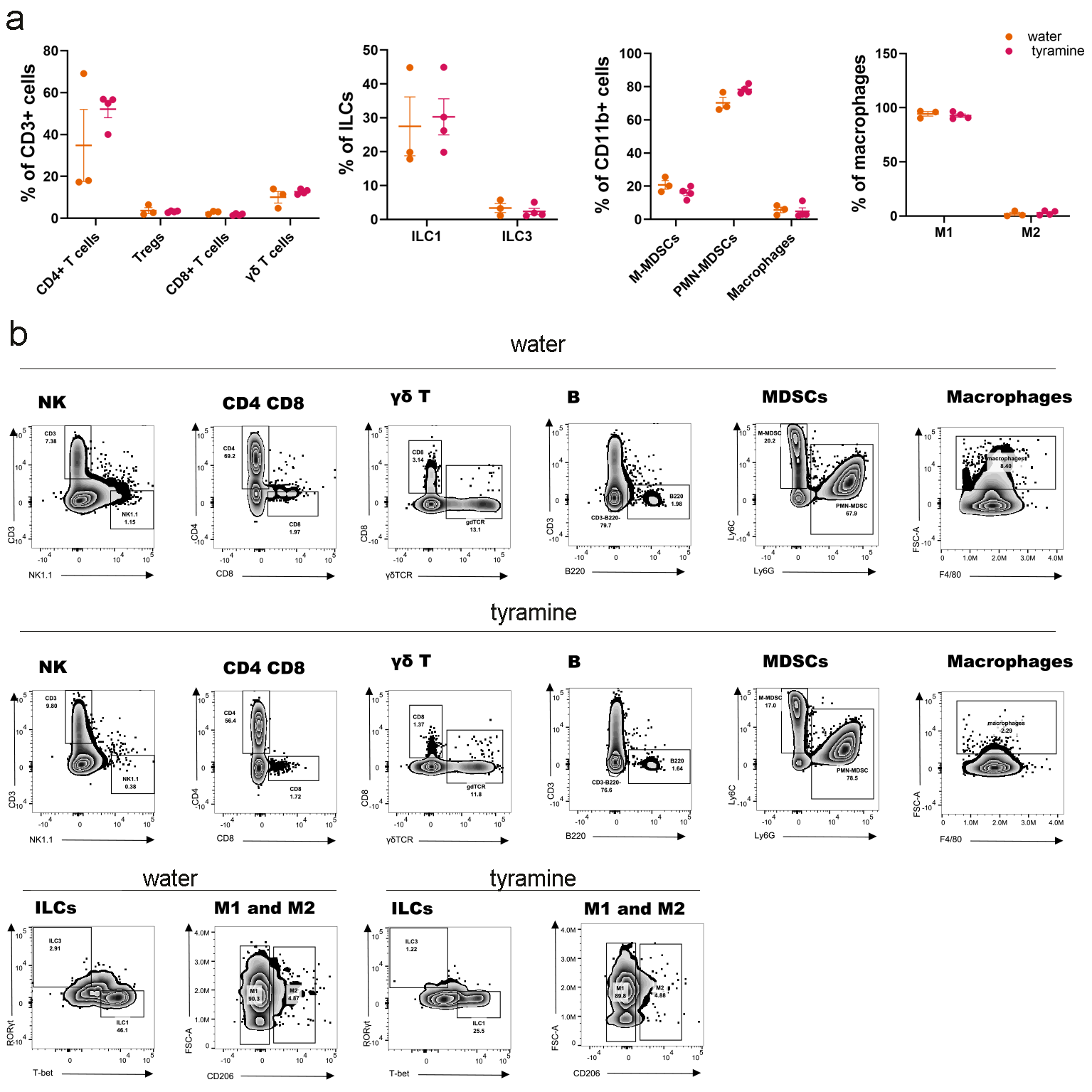


**Fig. S11** Similar frequencies of immune subsets in tumor bearing tyramine- and water- treated *Apc^Min/+^* mice. **a)** Percentages of lymphoid, myeloid, macrophages and CD11b+ immune populations in tumor infiltrating lymphocytes (TIL) in colon. Wilcoxon Rank Sum test was applied for between group comparisons followed by FDR method for adjusting p-values for multiple test correction. Orange, water-treated *Apc^Min/+^* mice; pink, tyramine-treated *Apc^Min/+^* mice. Data shown as means ± SEM. **b)** Representative flow cytometry zebra plots depicting NK, CD4+T, CD8+T, γδT, B, MDSCs, macrophages, ILC1, ILC2, M1 and M2 macrophages in tyramine- and water-treated groups.


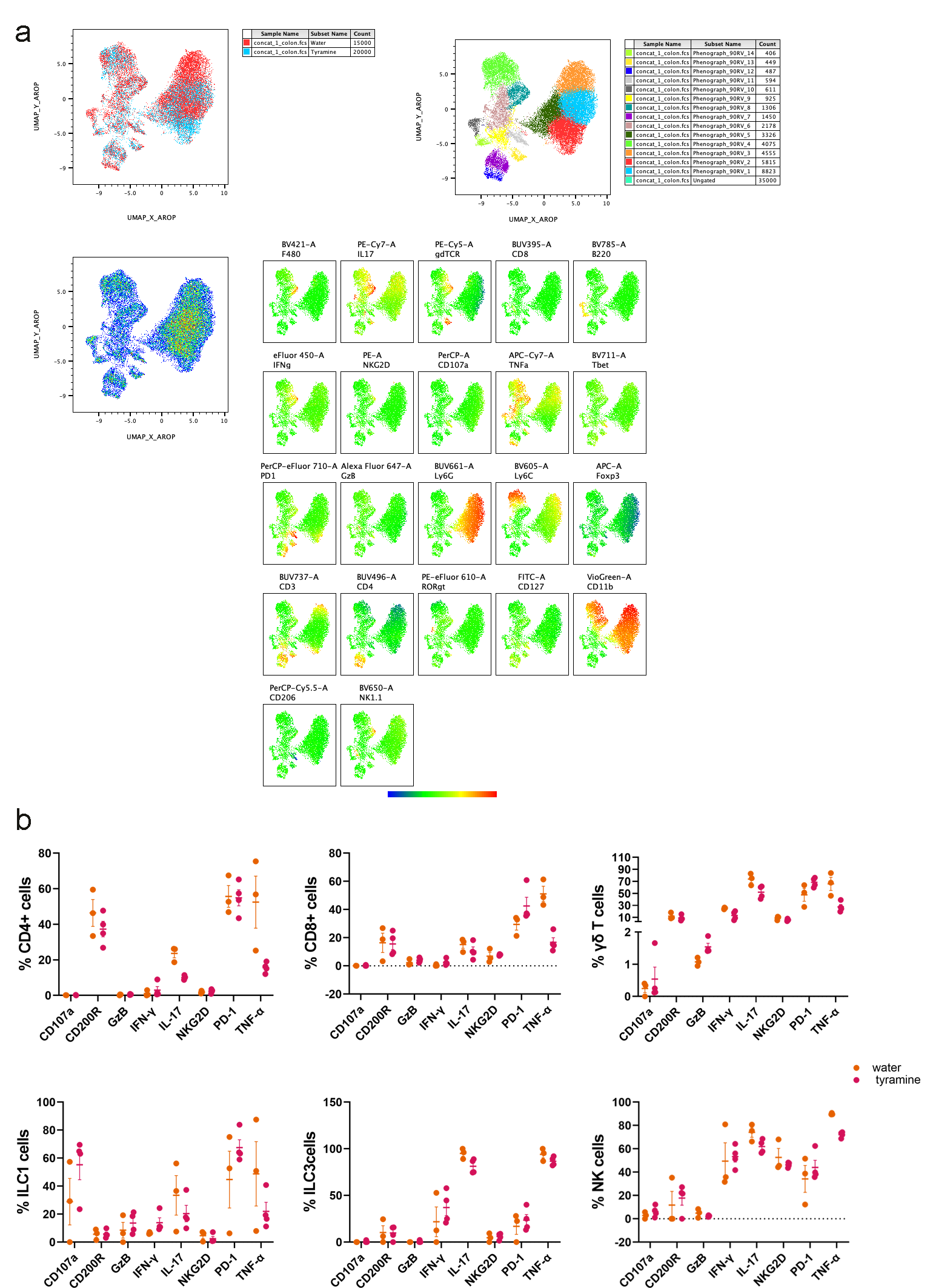


**Fig. S12** PMN-MDSCs segregate tyramine- and water- treatment groups in colonic TIL of *Apc^Min/+^* mice. **a)** UMAP analysis to identify distinct clusters unique to tyramine or water treatment. Phenograph analysis to determine the phenotype of the cell clusters and heatmaps with all parameters on all samples in the colon. **b)** Percentages of functional markers CD107a, CD200R, GzB, IFN-γ, IL-17, NKG2D, PD-1, and TNF-α in CD4+, CD8+, γδT, ILC1, ILC3 and NK cells**.** Orange, water-treated *Apc^Min/+^* mice; pink, tyramine-treated *Apc^Min/+^* mice. Wilcoxon Rank Sum test was applied for between group comparisons followed by FDR method for adjusting p-values for multiple test correction. Data shown as means±SEM.

**Fig. S13** Phenotypic characterization of tyramine-treated *Apc^Min/+^* and WT mice. *Apc^Min/+^* mice and respective WT littermate controls received daily doses of 3.2mM tyramine (n=13 for *Apc^Min/+^* mice and n=12 for WT mice) or water (n=12 for *Apc^Min/+^* mice and n=10 for WT mice) via oral gavage for a period of 49 days. Treatment started at 6 weeks of age and concluded at 13 weeks of age. **a)** Body weight, **b)** Food consumption and **c)** Water intake across the study among tyramine-treated *Apc^Min/+^* mice, untreated *Apc^Min/+^* mice, tyramine-treated WT and untreated WT mice. **d)** Spleen weight, **e)** Hematocrit, **f)** Liver weight, **g)** Gut length and **h)** Colon length at 13 weeks of age. **i)** Dysplasia scoring based on ileum gut rolls taken at study endpoint and representative H&E sections from tyramine-treated and untreated *Apc^Min/+^* mice. Scale bar 1mm for whole gut roll image. Square indicates the area of magnified image. Scale bar 250µm for magnified image. Orange, water-treated *Apc^Min/+^* mice; pink, tyramine-treated *Apc^Min/+^* mice; light blue, water-treated WT mice; purple, tyramine-treated WT mice. Data shown as means ± SEM. Two-way ANOVA with Tukey’s multiple comparisons test in a), mixed effects model in b) and c), ordinary one-way ANOVA with Tukey’s multiple comparisons test in d), g) and h), Kruskal-Wallis test with Dunn’s multiple comparisons test in e), f) and i). *, p<0.05; **, p<0.01, ****, p < 0.0001.


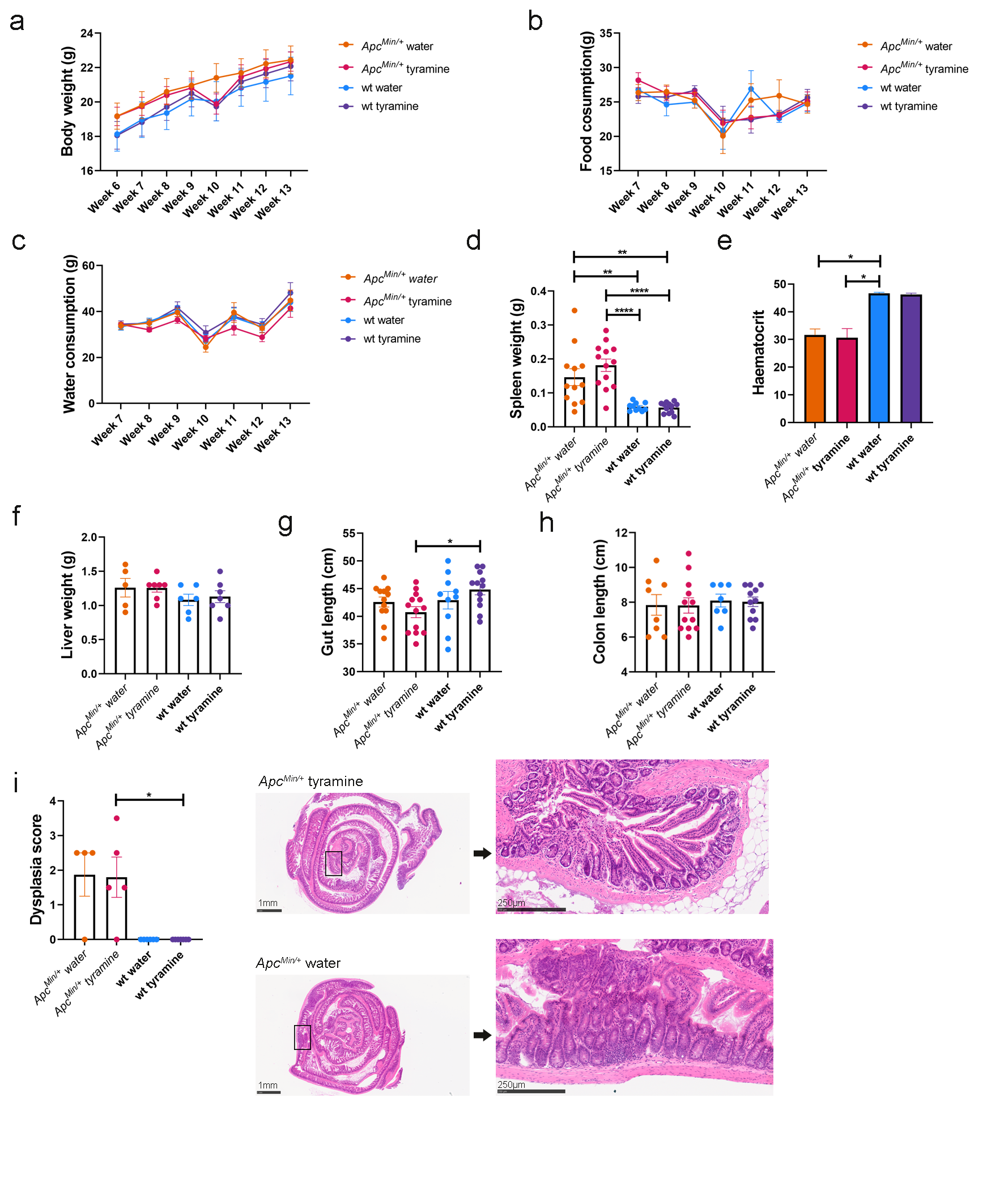


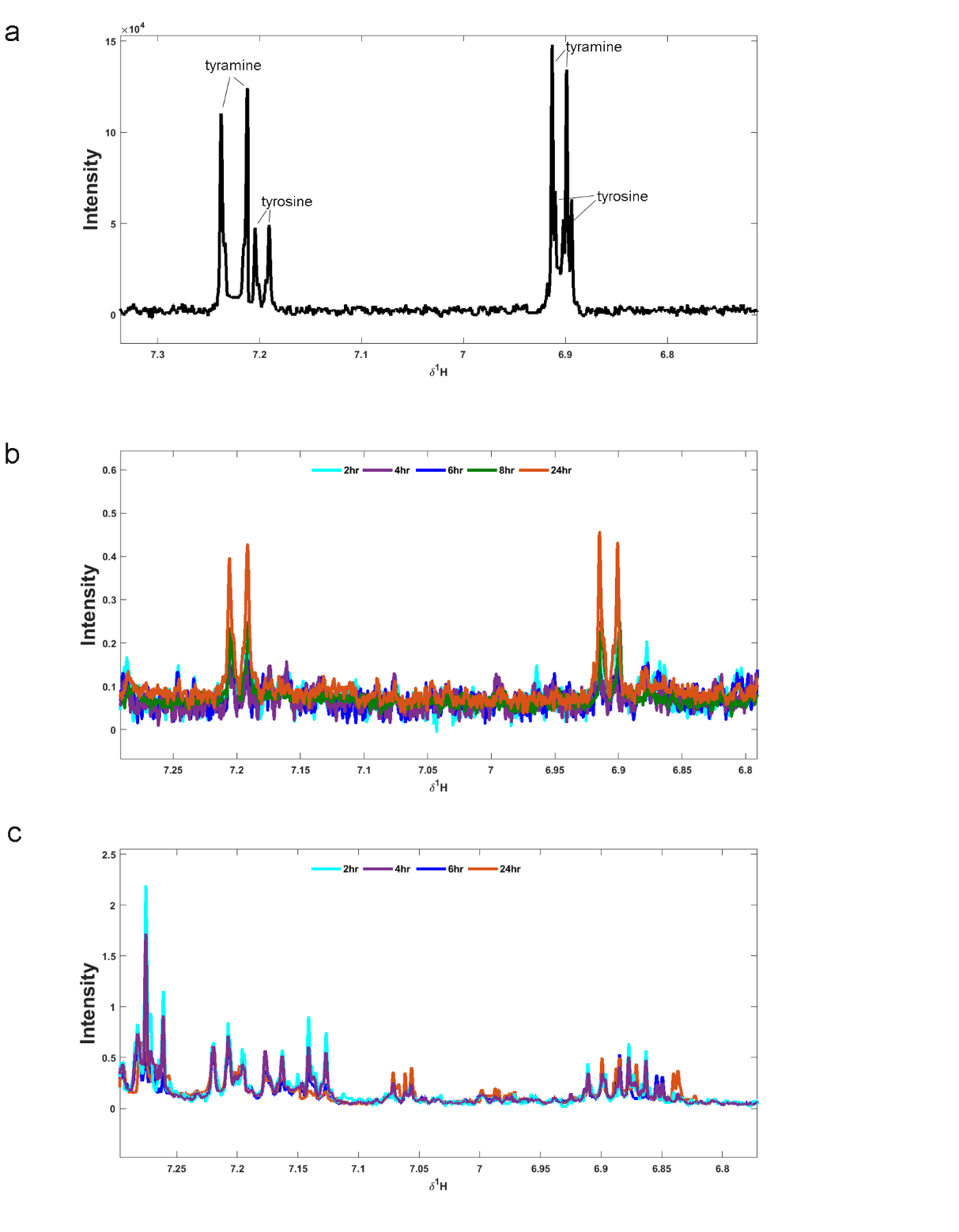


**Fig. S14** ^1^H NMR analysis of fecal water extracts and urinary samples for identification of tyramine. 8 mice (n=4 *Apc^Min/+^* and n=4 WT) were administered via oral gavage 3.2 mM tyramine, and feces and urine were collected at 2 hrs, 4 hrs, 6 hrs, 8 hrs, and 24 hrs post gavage. **a)** Magnified spectrum region showing a duplet peak of tyramine at 7.23 ppm and a duplet peak at 6.91 ppm in a buffer containing tyramine and tyrosine. Tyrosine is also shown, as it is very close to tyramine and is easily confused. Tyrosine has a duplet peak at 7.18 ppm and a duplet peak at 6.89 ppm. Magnified spectrum region in **b)** fecal water extract and **c)** in urine demonstrating the absence of tyramine peaks. A representative ^1^H NMR spectrum from one mouse is shown. Light blue, 2 hrs; purple, 4 hrs; dark blue, 6 hrs; orange, 24 hrs.

**References**

1. J. C. Arthur *et al.*, Intestinal inflammation targets cancer-inducing activity of the microbiota. *Science* **338**, 120-123 (2012).

2. O. Beckonert *et al.*, Metabolic profiling, metabolomic and metabonomic procedures for NMR spectroscopy of urine, plasma, serum and tissue extracts. *Nat Protoc* **2**, 2692-2703 (2007).

3. A. C. Dona *et al.*, Precision high-throughput proton NMR spectroscopy of human urine, serum, and plasma for large-scale metabolic phenotyping. *Anal Chem* **86**, 9887-9894 (2014).

4. K. A. Veselkov *et al.*, Recursive segment-wise peak alignment of biological (1)h NMR spectra for improved metabolic biomarker recovery. *Anal Chem* **81**, 56-66 (2009).

5. F. Dieterle, A. Ross, G. Schlotterbeck, H. Senn, Probabilistic quotient normalization as robust method to account for dilution of complex biological mixtures. Application in 1H NMR metabonomics. *Anal Chem* **78**, 4281-4290 (2006).

6. J. C. Lindon, J. K. Nicholson, Spectroscopic and statistical techniques for information recovery in metabonomics and metabolomics. *Annu Rev Anal Chem (Palo Alto Calif)* **1**, 45-69 (2008).

7. B. M. Beckwith-Hall *et al.*, Application of orthogonal signal correction to minimise the effects of physical and biological variation in high resolution 1H NMR spectra of biofluids. *Analyst* **127**, 1283-1288 (2002).

8. O. Cloarec *et al.*, Statistical total correlation spectroscopy: an exploratory approach for latent biomarker identification from metabolic 1H NMR data sets. *Anal Chem* **77**, 1282-1289 (2005).

9. K. Haug *et al.*, MetaboLights: a resource evolving in response to the needs of its scientific community. *Nucleic Acids Res* **48**, D440-D444 (2020).

10. W. J. Chae, A. L. Bothwell, IL-17F deficiency inhibits small intestinal tumorigenesis in ApcMin/+ mice. *Biochem Biophys Res Commun* **414**, 31-36 (2011).

11. M. C. Myzak, W. M. Dashwood, G. A. Orner, E. Ho, R. H. Dashwood, Sulforaphane inhibits histone deacetylase in vivo and suppresses tumorigenesis in Apc-minus mice. *FASEB J* **20**, 506-508 (2006).

12. Y. Li *et al.*, Gut microbiota accelerate tumor growth via c-jun and STAT3 phosphorylation in APCMin/+ mice. *Carcinogenesis* **33**, 1231-1238 (2012).

13. K. Bugda Gwilt *et al.*, Actions of Trace Amines in the Brain-Gut-Microbiome Axis via Trace Amine-Associated Receptor-1 (TAAR1). *Cellular and Molecular Neurobiology* **40**, 191-201 (2019).

14. D. M. Tchercansky, C. Acevedo, M. C. Rubio, Studies of tyramine transfer and metabolism using an in vitro intestinal preparation. *J Pharm Sci* **83**, 549-552 (1994).
